# Fuzzy C-Means clustering and LSTM-based magnitude prediction of earthquakes in the Aegean region of Türkiye

**DOI:** 10.1038/s41598-025-07538-w

**Published:** 2025-09-30

**Authors:** Badr Aloraini, Hatice Oncel Cekim, Hatice Nur Karakavak, Gamze Ozel

**Affiliations:** 1 Department of Mathematics, College of Science and Humanities, Shaqra University, 11961 Shaqra, Saudi Arabia; 2 Department of Statistics, Hacettepe University, 06800 Ankara, Turkey

**Keywords:** Fuzzy C-Means clustering, Gumbel distribution, Long-short term memory, Earthquake magnitudes, Environmental sciences, Natural hazards, Solid Earth sciences

## Abstract

Türkiye is highly susceptible to earthquakes due to its active tectonic structure and the presence of major fault lines. The accurate estimation of earthquake magnitudes is essential for effective risk mitigation and structural resilience. This study proposes an integrated methodology combining clustering, statistical modeling, and deep learning techniques for the analysis and forecasting of earthquake magnitudes. Initially, earthquakes are classified into three distinct regions using the Fuzzy C-Means (FCM) clustering algorithm. For each region, statistical distributions are applied to characterize magnitude behavior. Subsequently, the Long Short-Term Memory (LSTM) model is used to predict future earthquake magnitudes. The joint application of these three methods provides a comprehensive framework for regional seismic analysis. The findings suggest that the Gumbel distribution offers the best fit for modeling return periods of earthquakes with magnitudes greater than Mwg 5.18, where Mwg denotes the global moment magnitude. Estimated return periods range from 2.56 to 3.63 years in the first region, 2.53 to 3.55 years in the second region, and 2.71–4.22 years in the third region, based on probability levels between 25 and 95%. The LSTM model forecasts that the third region is likely to experience relatively stronger seismic activity, with maximum magnitudes ranging from 2.4 to 6.5 between October 2021 and March 2029. For the same period, expected magnitudes in the first and second regions range from 2.0 to 5.7. These forecasts are supported by model performance metrics that confirm the projected magnitudes are within an acceptable and reliable range of accuracy for medium-term seismic forecasting.

## Introduction

Earthquakes are powerful natural disasters that can cause severe destruction, affecting lives and property worldwide. Analyzing the impacts of earthquakes is crucial, as seismic events can damage infrastructure and lead to long-term economic consequences^1^. Türkiye faces significant earthquake risks, especially in the northwestern and western regions, where both the North Anatolian Fault Zone (NAFZ) and the Western Anatolian Fault Zone (WAFZ) contribute to destructive seismic activity^2^. The westward movement of the Anatolian plate increases the earthquake threat in cities near the NAFZ and active fault lines in the Aegean region. Istanbul, a major city located on the NAFZ, experienced the devastating August 17, 1999 earthquake (magnitude 7.28), which resulted in over 17,000 deaths and widespread damage^3^. Southern cities such as Düzce and Bursa are also vulnerable, as demonstrated by the November 12, 1999 Düzce earthquake (magnitude 7.06)^4^. Provinces like Balıkesir and Çanakkale were affected by the Yenice-Gönen earthquake (magnitude 7.28) in 1953. The Aegean city of İzmir has experienced several strong earthquakes, including the June 6, 1970 event (magnitude 7.06), which resulted in 1,086 deaths, and the October 30, 2020 earthquake (magnitude 6.73), which triggered aftershocks and a tsunami^5^. These events underscore the ongoing seismic threat and the urgent need for preparedness in these high-risk areas.

Western Anatolia is one of the most active seismotectonic regions in Türkiye, characterized by frequent historical and recent earthquakes. This high-risk nature of the region necessitates detailed and multidisciplinary studies, particularly concerning the safety of settlements and the planning of engineering structures. In recent years, many studies have contributed to a better understanding of ground motion behavior and to improving the accuracy of earthquake impact predictions. For instance, Bayrak and Çoban^6^ examined ground motion parameters and Coulomb stress changes related to the 2019 Bozkurt (Denizli) earthquake, offering insights into seismic hazard. Paradisopoulou et al.^7^ used stress transfer models to explain the timing and spatial distribution of large earthquakes in Western Türkiye. Bayrak and Bayrak^8^ assessed seismic hazard across Western Anatolia by combining historical and instrumental data. Çoban and Sayıl^9^ estimated the probabilities of major potential earthquakes in the Hellenic Arc using statistical distribution models. Tepe et al.^10^ prepared a new local and updated historical earthquake catalog for Izmir and its immediate surroundings by examining many original sources, records and old international earthquake catalogs in addition to the existing national catalogs used in seismicity studies in Türkiye and determining new geological, seismological and environmental data. Uzelli et al.^11^ investigated changes in groundwater levels following the 2020 Samos earthquake in İzmir, highlighting the relationship between earthquakes and hydrogeology. Mozafari et al.^12^ identified seismic activity over the past 16,000 years through 36Cl dating of carbonate fault surfaces. Finally, Ermiş and Curebal^13^, focused on seismic activity prediction by applying decision tree models by examining earthquake records in the Izmir province in western Türkiye. These studies clearly emphasize the importance of closely monitoring Western Anatolia in terms of seismicity.

Earthquake prediction remains one of the most challenging problems in geosciences and has been addressed from various theoretical and practical perspectives. Classical approaches include deterministic models that attempt to forecast earthquakes based on stress accumulation, fault interactions, and physical precursors^14,15^. However, such models are often limited by the complex and chaotic nature of tectonic processes. As an alternative, statistical and probabilistic models such as Poisson, renewal, and time-dependent hazard models have been widely adopted^16–18^. These models aim to describe the frequency and magnitude of earthquakes over time and are typically used in seismic hazard maps and risk assessments. Despite their usefulness, they often assume spatial and temporal stationarity and struggle to capture evolving seismic behaviors in heterogeneous regions like Western Anatolia. To overcome these limitations, recent studies have increasingly explored the potential of machine learning (ML) and deep learning (DL) methods in earthquake prediction. Algorithms such as Support Vector Machines (SVM), Random Forests, and especially neural networks like Recurrent Neural Networks (RNN) and Long Short-Term Memory (LSTM) networks have demonstrated promising results in modeling nonlinear temporal dependencies and classifying seismic patterns^19–21^. For instance, Wang et al.^22^ used LSTM models to predict magnitude time series in China, while Aryal et al.^23^ applied LSTM to forecast seismic activity in Nepal and its surrounding regions. Other researchers^24,25^ combined LSTM with optimization techniques such as genetic algorithms or attention mechanisms to improve forecast accuracy. In parallel, clustering and unsupervised learning techniques have been employed to identify latent structures in earthquake catalogs. These include hierarchical clustering, k-means, and fuzzy clustering methods such as Fuzzy c-Means (FCM)^26,27^, which allow for the detection of overlapping seismic zones an important consideration in tectonically complex regions.

Statistical analyses are also used to better understand how and why earthquakes occur^16,17,28^. Cluster analysis aims to identify distinct groups within a dataset, where observations in the same cluster share similar characteristics. In seismology, cluster analysis is often applied to classify earthquakes based on features such as magnitude and depth^26,27^. The FCM algorithm allows data points to belong to multiple clusters with varying degrees of membership. In this study, the FCM method is preferred over traditional clustering techniques due to its advantages in handling the complex nature of earthquake data, which often involves uncertainty and lacks clearly defined boundaries. This method contributes significantly to earthquake prediction, classification, and hazard assessment by uncovering patterns and influential factors in seismic activity. In addition, statistical distributions of earthquake magnitudes have been widely used to evaluate seismicity across different regions. The Gumbel distribution is a commonly applied model in earthquake studies, as it focuses on extreme magnitudes to assess seismic risk. Although Kaila and Narain^29^ criticized this approach for neglecting smaller events, Bath^30^ argued that focusing on larger earthquakes is advantageous, as their magnitudes are more accurately recorded and have greater impact. Compared to other models such as the exponential and Weibull distributions, the Gumbel distribution is particularly effective for analyzing extreme seismic events.

The LSTM method is distinguished by its ability to effectively handle the irregular structure of earthquake magnitudes through hyper parameter tuning and to manage complex, multidimensional data. These features facilitate efficient analysis of earthquake data, improve the accuracy of predictions, and support the advancement of earthquake risk management. Due to its strong learning capacity, LSTM can simultaneously learn complex patterns and temporal dependencies. This capability makes it widely used for modeling multidimensional and complex processes in earthquake data. Wang et al.^22^ utilized LSTM networks to estimate earthquake magnitudes, demonstrating the effectiveness of this method in capturing the intricate dynamics of complex time series data. Jena et al.^31^ applied the LSTM model in combination with Geographic Information Systems (GIS) techniques to assess earthquake safety across India. In a more recent study, Berhich et al.^32^ used earthquake data from Japan spanning 1900 to 2021 to estimate magnitudes and compared the results with those obtained from other deep learning models. Sadhukhan et al.^33^ examined LSTM, bidirectional LSTM (Bi-LSTM), and a converter model to forecast earthquake magnitudes in three different seismic regions. Aryal et al.^34^ employed the LSTM model to predict earthquake magnitudes in Nepal and its neighboring regions and found that the model effectively captured overall seismic trends and maintained consistent performance over time. Ye et al.^24^ developed an earthquake forecasting system that combines the Elite Genetic Algorithm, used for feature selection, with the LSTM model, which captures the impact of past events on future predictions. The study concluded that this combined system improved prediction performance compared to other models and the standard LSTM approach. As demonstrated by these studies, LSTM is selected as the preferred deep learning method in the present study, owing to its proven success in numerous applications, particularly in earthquake magnitude estimation.

Despite these developments, comprehensive hybrid frameworks that simultaneously incorporate spatial clustering, magnitude modeling using extreme value distributions, and deep learning–based forecasting are still scarce in the literature. The present study aims to fill this gap by proposing an integrated methodology for localized and data-driven earthquake prediction, combining unsupervised classification (FCM), extreme value analysis (Gumbel distribution), and temporal magnitude forecasting (LSTM) tailored for the Western Anatolian seismic zone. Although numerous studies have addressed the seismicity and earthquake hazard of Türkiye^2,35–37^, particularly in the Aegean and Western Anatolian regions, many rely on conventional methodologies such as tectonic zonation, Gutenberg–Richter-based recurrence models, and probabilistic seismic hazard mapping^38,39^. While these methods provide valuable insights, they often assume regional homogeneity and do not account for the spatial heterogeneity, nonlinear dynamics, and temporal evolution of earthquake occurrence. Moreover, traditional models rarely incorporate data-driven clustering or modern predictive techniques, limiting their capacity to identify evolving patterns in regional seismic activity. To address these limitations, the present study proposes a novel hybrid framework that integrates FCM clustering^40^, Gumbel-based extreme value modeling^41^, and LSTM neural networks^42^. This approach enables the objective delineation of seismic sub-regions based on observed data, the characterization of magnitude extremes for each cluster, and the forecasting of regional seismic trends based on historical magnitude patterns. By combining unsupervised learning, statistical distribution modeling, and deep learning, this study advances beyond traditional hazard assessments and contributes a flexible, adaptive, and predictive tool for localized seismic risk evaluation.

**Fig. 1:**
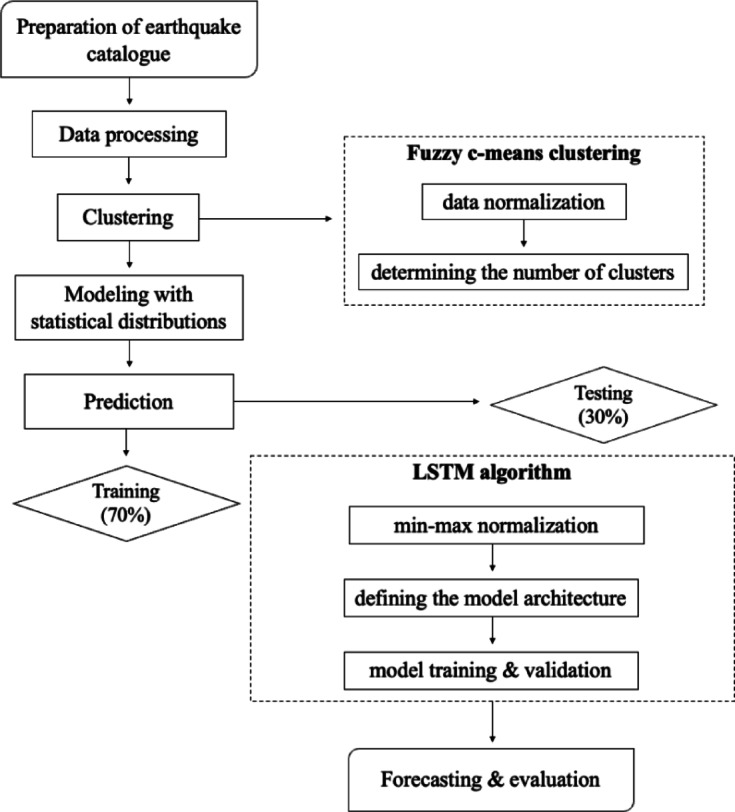
Schematic diagram of the applied methodology.

The main objective of this study is to divide the Aegean region into sub-regions based on seismic events that occurred between 1970 and 2021, to analyze the distributional characteristics of these sub-regions, and to predict the maximum earthquake magnitudes that may occur in future periods. In this context, the region is initially partitioned into three sub-regions using the FCM method, which is robust to anomalies in earthquake data. By identifying the statistical distributions and characteristics of each sub-region, the suitability of the clustering results was evaluated. Subsequently, the LSTM method, which has a strong learning and adaptation mechanism based on historical seismic data, was applied to forecast future earthquake magnitudes in each sub-region. Then, the Aegean region was successfully divided into appropriate sub-regions, and potential seismic activity was analyzed in detail for the first time using this combined methodology. The structure of the paper is presented in Fig. 1. Section "Study area" provides a detailed overview of the study area and the earthquake catalog. Section "Methodology" explains the methodology, including FCM, LSTM, and statistical distribution models. Section "Results and discussion" presents the results, and Section "Conclusion" concludes with a summary of key findings and their implications.

## Study area

**Fig. 2:**
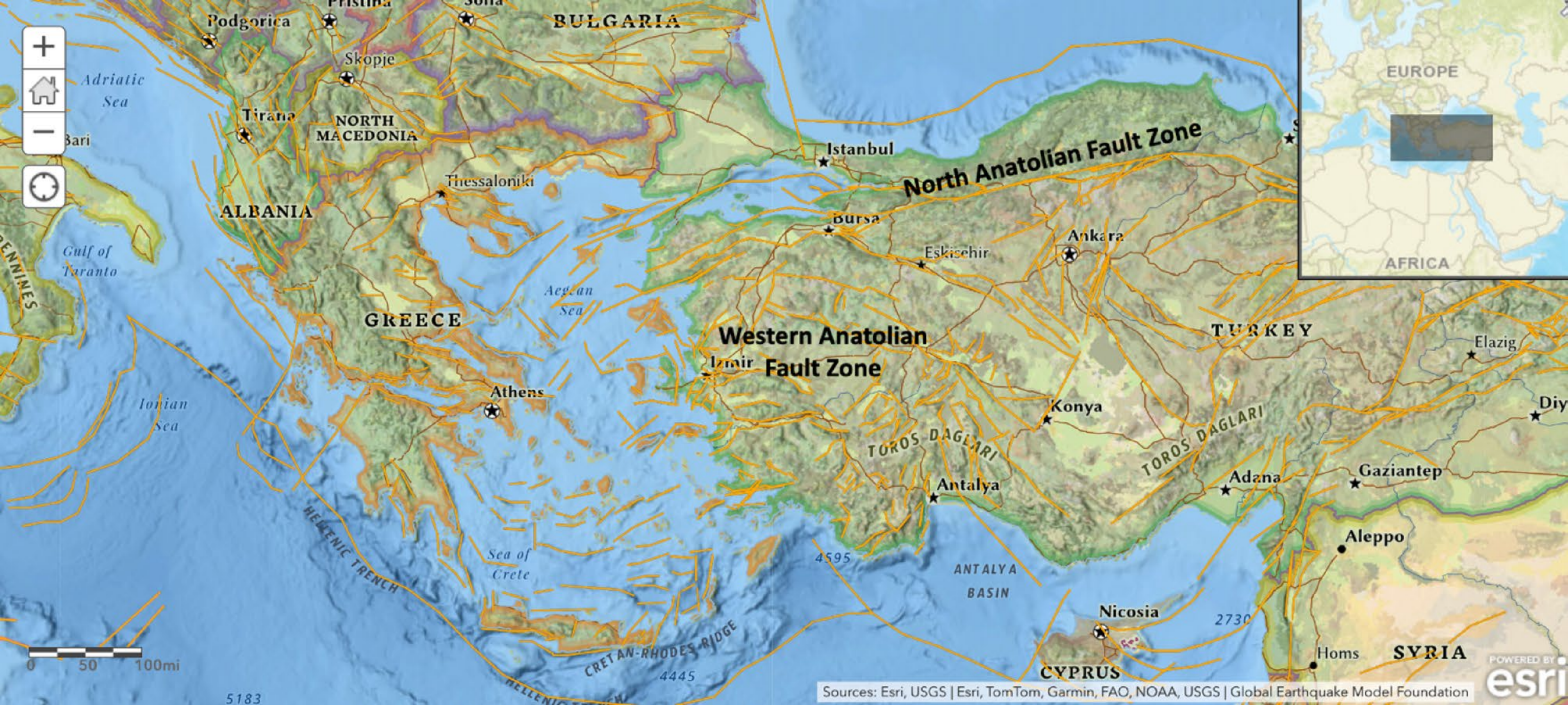
Study area and active fault lines within the Western Anatolian extensional regime (ArcGIS Online, (Esri, accessed 2025); https://www.arcgis.com/).

Türkiye is located at the intersection of Western Asia and Eurasia, within the seismically active Alpine-Himalayan belt^43^. Its complex geology and geodynamic setting give rise to numerous active fault lines^44,45^. The Aegean Region, situated near the Gediz Graben system, is one of the most seismically active areas in Türkiye, with a long history of frequent earthquakes^2,46,47^. The region’s tectonic complexity includes several distinct fault lines, and Türkiye has been classified into four neotectonic provinces by Şengör^48,49^ and Emre et al.^50^. This study focuses on earthquakes with a moment magnitude of Mwg ≥ 2.43 that occurred in the Aegean Region between 1970 and 2021. Figure 2 presents a map illustrating the active fault zones.

The dataset used in this study was compiled by integrating earthquake records from three major sources: the Disaster and Emergency Management Presidency Earthquake Department (AFAD-DDA), the Kandilli Observatory and Earthquake Research Institute (KOERI), and the catalog prepared by Tan^51^. Within the study area, these catalogs respectively documented 202,080, 13,950, and 252,594 earthquakes between 1900 and 2021. To construct a unified and comprehensive dataset, events were matched across catalogs using two criteria: (i) a time difference of less than 3 min and (ii) a spatial difference of less than 0.5 degrees in both latitude and longitude. A strong correlation was observed, particularly between the AFAD-DDA and Tan^51^ catalogs, especially for events with magnitudes greater than Mwg ≥ 2.43. Following the matching process, unmatched events and missing records were incorporated into the AFAD-DDA dataset, and all magnitude values were standardized using moment magnitude conversion equations to ensure consistency across sources.

**Fig. 3:**
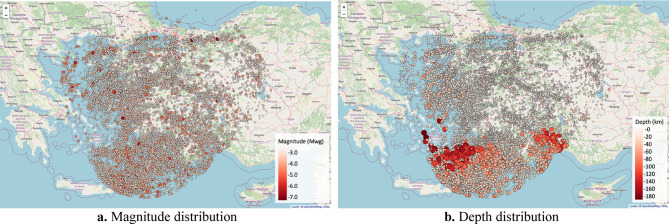
Spatial distributions of earthquake magnitudes (**a**) and depths (**b**) in the declustered catalog (Mwg ≥ 2.43) for the period 1970–2021 (R version 4.3.1; https://www.r-project.org/).

To avoid statistical bias caused by dependent events such as aftershocks, foreshocks, and earthquake swarms, the compiled catalog was declustered using the Reasenberg algorithm^52^, which identifies and removes time–space-magnitude correlated event clusters. This procedure was implemented through the ZMAP software environment^53^, which is widely used in seismological research for catalog cleaning and event independence analysis. The declustering algorithm identified 5,284 dependent event clusters, resulting in the removal of 30,107 earthquakes, predominantly short-interval aftershocks. The remaining catalog consisted of 16,503 independent seismic events, spanning from January 1970 to September 2021, which formed the basis of the subsequent clustering, statistical modeling, and forecasting procedures. By removing temporally and spatially correlated events, the declustering process ensured that the dataset would represent the background seismicity of the region. This is crucial for the validity of clustering and modeling steps, as the presence of dependent events would artificially inflate seismic density in certain locations and lead to misleading interpretations of seismic hazard. The spatial distribution of moment magnitudes (Mwg) and depths (km) for the declustered dataset is presented in Fig. 3.

Various empirical relationships have been employed to convert different magnitude types to moment magnitude (Mw). In this study, the conversion formulas proposed by Kadirioğlu and Kartal^54^, Pınar et al.^55^, Tan^51^, and Scordilis^56^ were considered. The primary reason for selecting these relations is that they were derived from the same or similar earthquake catalogs used in this study (e.g.^51,55^) and are widely accepted for magnitude conversion in the region (e.g.^56^). The equations representing the original magnitude-to-moment magnitude (Mw) relationships taken from Kadirioğlu and Kartal^54^ and used for homogenizing the earthquake catalog are provided below:

$$\begin{aligned} M_{w} = & 0.5716\left( { \pm 0.024927} \right)M_{s} + 2.4980\left( { \pm 0.117197} \right); 3.4 \le M_{s} \le 5.4 \\ M_{w} = & 0.8126\left( { \pm 0.034602} \right)M_{s} + 1.1723\left( { \pm 0.208173} \right); M_{s} \ge 5.5 \\ M_{w} = & 1.0319\left( { \pm 0.025} \right)M_{b} + 0.0223\left( { \pm 0.130} \right); 3.9 \le M_{b} \le 5.8 \\ M_{w} = & 0.7947\left( { \pm 0.033} \right)M_{d} + 1.3420\left( { \pm 0.163} \right); 3.5 \le M_{d} \le 5.0 \\ M_{w} = & 0.8095\left( { \pm 0.031} \right)M_{l} + 1.3003\left( { \pm 0.154} \right); 3.3 \le M_{l} \le 5.3 \\ \end{aligned}$$

The conversion relationships between original magnitude types and moment magnitude (Mw) proposed by Pınar et al.^55^ are given below:

$$\begin{aligned} M_{w} = & 0.8466\left( { \pm 0.0514} \right)M_{s} + 1.2157\left( { \pm 0.01304} \right) \\ M_{w} = & 0.8998\left( { \pm 0.0637} \right)M_{b} + 0.5548\left( { \pm 0.0168} \right) \\ M_{w} = & 0.9321\left( { \pm 0.0637} \right)M_{d} + 0.3353\left( { \pm 0.0168} \right) \\ M_{w} = & 0.9451\left( { \pm 0.0406} \right)M_{l} + 0.3163\left( { \pm 0.0103} \right) \\ \end{aligned}$$

The original magnitude-to-moment magnitude (Mw) conversion relationships proposed by Tan^51^ are as follows:

$$\begin{aligned} M_{w} = & 0.827\left( { \pm 0.05} \right)M_{s} + 1.181\left( { \pm 0.21} \right), 4.0 \le M_{s} \le 7.7 \\ M_{w} = & 1.043\left( { \pm 0.02} \right)M_{b} - 0.08\left( { \pm 0.08} \right), 4.0 \le M_{b} \le 7.0 \\ M_{w} = & 1.111\left( { \pm 0.03} \right)M_{d} - 0.459\left( { \pm 0.14} \right), 2.8 \le M_{d} \le 7.3 \\ M_{w} = & 1.017\left( { \pm 0.02} \right)M_{l} - 0.012\left( { \pm 0.07} \right), 2.8 \le M_{l} \le 7.2 \\ \end{aligned}$$

The original magnitude-to-moment magnitude (Mw) conversion relationships proposed by Scordilis^56^, and used for homogenizing the earthquake catalog, are presented below:

$$\begin{aligned} M_{w} = & 0.67\left( { \pm 0.005} \right)M_{s} + 2.07\left( { \pm 0.03} \right), 3.0 \le M_{s} \le 6.1 \\ M_{w} = & 0.99\left( { \pm 0.02} \right)M_{s} + 0.08\left( { \pm 0.13} \right), 6.2 \le M_{s} \le 8.2 \\ M_{w} = & 0.85\left( { \pm 0.04} \right)M_{b} + 1.03\left( { \pm 0.23} \right), 3.5 \le M_{b} \le 6.2 \\ \end{aligned}$$

However, the Mw scale is available for large earthquakes (> 7.5), it has not been validated globally for magnitudes below 7.5 and is not suitable for use. In addition, the Mw scale is based on surface waves and may not be valid for all earthquake depths. To address these shortcomings, Das et al.^57^ introduced the Das Magnitude Scale (Mwg) using global data covering 25,708 earthquakes between 1976 and 2006. Mwg uses body wave magnitude instead of surface waves and is applicable to all depths, making it more suitable for a wider range of earthquakes. Mwg is a more accurate measure of energy release than Mw and provides a better representation of earthquake power^58^. Therefore, after obtaining the Mw magnitudes in the study, the seismic moment (Mo) proposed by Hanks and Kanamori^59^ was first calculated and then the Mwg scale was calculated to be used in the analyses throughout the study. The equations used are as follows, respectively:

$$log{M}_{o}=\left({M}_{w}+10.7\right)*\frac{3}{2},$$

$${M}_{wg}=\frac{log{M}_{o}}{1.36}-12.68 \ .$$

**Fig. 4:**
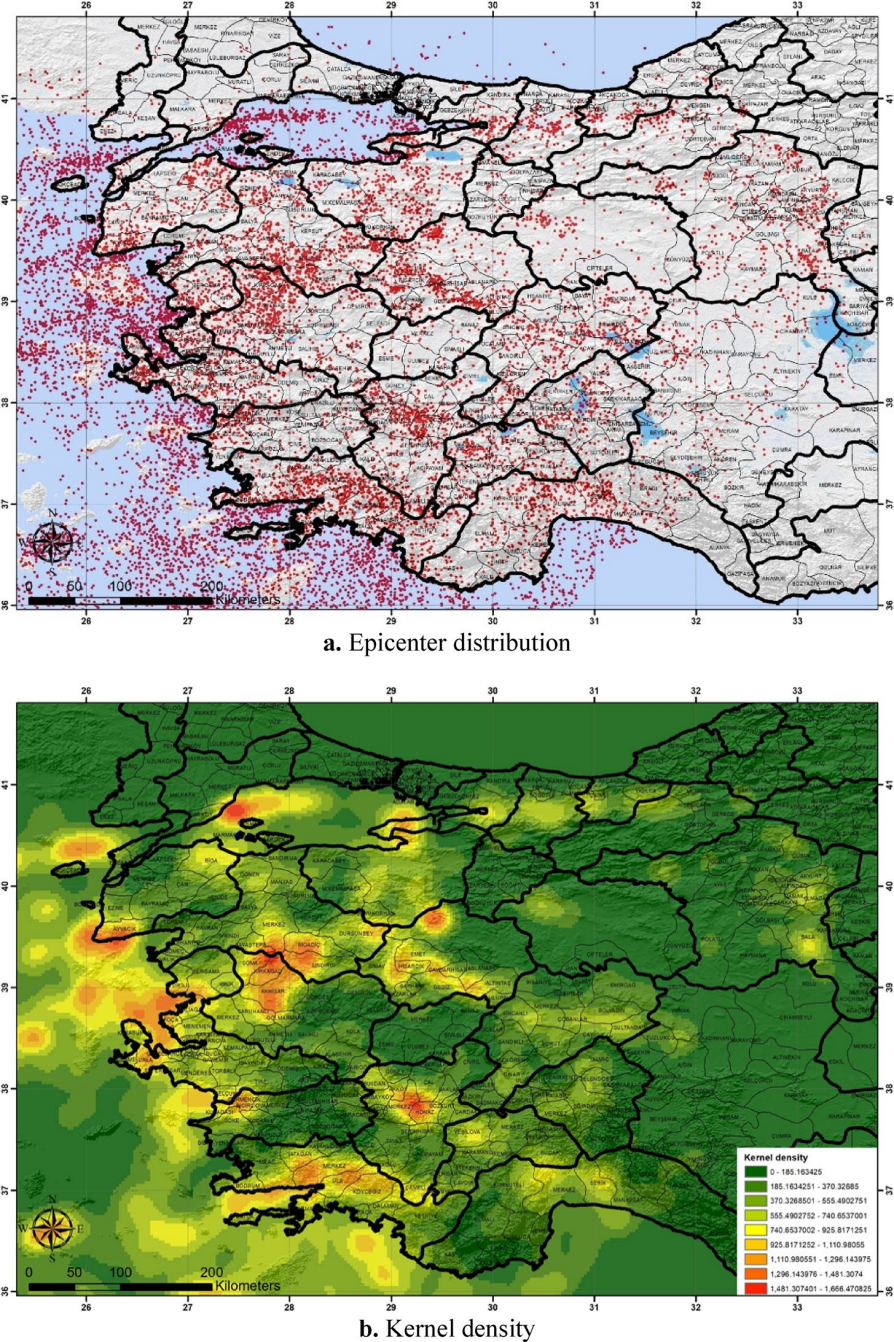
Map of the epicenter distribution (**a**) and Kernel density (**b**) of the non-clustered catalog of earthquakes with Mwg ≥ 2.4 (ArcGIS; https://www.arcgis.com/)^60^.

To visually represent the epicentral distribution and Kernel density of the earthquakes in the declustered earthquake catalog, maps were generated using ArcGIS and are showcased in Fig. 4a,b respectively. These steps were pivotal in ensuring the compilation of a reliable and declustered earthquake catalog for the study.

## Methodology

### FCM algorithm

The Fuzzy C-Means (FCM) method is a widely used approach in multivariate statistical analysis for partitioning data into distinct subsets. Unlike hard clustering methods, FCM allows each data point to belong to multiple clusters with varying degrees of membership, providing a more flexible and realistic representation of the data structure. In this method, the standardized Euclidean distance is commonly used as the distance metric^61^. A crucial initial step in the FCM algorithm is determining the appropriate number of clusters^62^. Several algorithms have been proposed in the literature to identify the optimal number of clusters in FCM. Two widely used methods for this purpose are the Silhouette score and the Elbow criterion^63^. The Silhouette score evaluates how well each data point fits its assigned cluster by considering both cohesion and separation. A value close to 1 indicates strong clustering performance, while a value near -1 suggests poor clustering. Higher Silhouette scores reflect better model suitability for the dataset. The Elbow criterion, on the other hand, analyzes the sum of squared errors (SSE) across different values of *k* (number of clusters). By plotting SSE against *k*, the “elbow” point where the rate of decrease sharply changes helps determine the optimal number of clusters^64,65^. Using both the Silhouette score and the Elbow criterion, the optimal number of clusters and clustering performance can be effectively assessed. This combined approach increases the flexibility and reliability of cluster analysis in multivariate contexts. After choosing the number of clusters (${c}_{i}, i=\text{1,2},\dots ,c),$ the cluster centers are obtained by

1 $${c}_{i}=\frac{{\sum }_{j=1}^{N}{u}_{ij}^{m}{X}_{j}}{{\sum }_{j=1}^{N}{u}_{ij}^{m}},$$

where *N* is the number of observations, *m* is called the turbidity coefficient that any real number greater than 1. Here, ${u}_{ij}$ represents the membership degree of ${X}_{i}$ in *j*^th^ cluster and it is given by

2 $${u}_{ij}=\frac{1}{{\sum }_{k=1}^{c}{\left(\frac{{d}_{ij}}{{d}_{kj}}\right)}^{2/(m-1)}} .$$

Here, ${d}_{ij}$ is the Euclidean distance between the cluster centers and the data as ${d}_{ij}=\Vert {c}_{i}-{X}_{j}\Vert$. Then, the objective function is given by

3 $$J\left(U, {c}_{1}, \dots , {c}_{c}\right)=\sum_{i=1}^{c}{J}_{i}=\sum_{i=1}^{c}\sum_{j}^{n}{u}_{ij}^{m}{d}_{ij}^{2}.$$

**Fig. 5:**
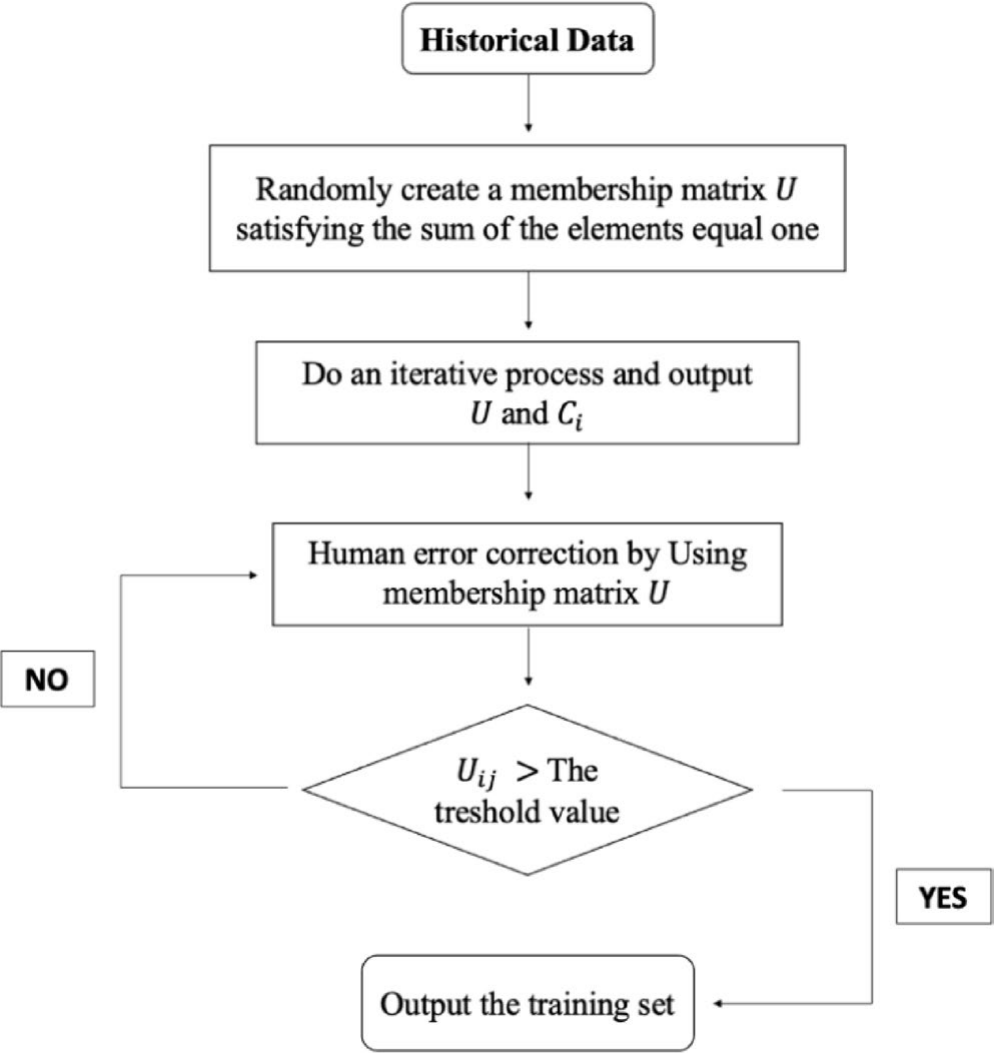
Flowchart of the FCM Algorithm^66^.

If the amount of change is small enough, the algorithm ends. The flowchart of the FCM algorithm is presented in Fig. 5.

### Statistical distributions

The most widely used formula for determining the magnitude-frequency relationship in earthquakes is the Gutenberg-Richter formula^67^. The Gutenberg-Richter formula is defined as

4 $$LogN\left(M\right)=a-bM,$$

where *N(M)* is the number of earthquakes equal or larger than *M* magnitude. The constants (*a*) and (*b*) in the magnitude-frequency relation represent key parameters. The average yearly seismic activity index is determined by the value of (*a*), which is influenced by both the observation period and the level of seismic activity. Parameter (*b*) governs the slope of the linear function. Regional-scale estimates of the *b*-value typically fall within the range of 0.5 to 1.5^68^. Besides, the average regional scale estimates of the *b*-value tend to hover around 1^69^. The *b*-value serves as an indicator of different tectonic sources within the given area^70^. The *a*-value is directly computed as the intercept in the Gutenberg-Richter relationship. By leveraging the parameters in this formula, probabilities of earthquake occurrences and return periods can be estimated using statistical distribution models such as Poisson, Gumbel, and others^71,72^.

The normal distribution is a symmetrical probability distribution centered around the mean and primarily used to model continuous and naturally occurring phenomena. Although it is not typically the most appropriate model for characterizing earthquake magnitudes—particularly due to the skewed and heavy-tailed nature of seismic data—it remains important as a baseline reference distribution when comparing the fit of alternative statistical models. In the context of seismology, the normal distribution can provide a useful benchmark for identifying deviations from symmetry and assessing the appropriateness of alternative models such as Gumbel, Log-Normal, or Weibull, which are more suitable for modeling extreme events. While earthquakes do not follow a strictly Gaussian behavior, understanding the behavior of the normal distribution enables researchers to assess whether the observed data exhibit asymmetry or heavier tails, which is critical for selecting accurate seismic hazard models. The cumulative distribution function (cdf) and probability density function (pdf) of the normal distribution are given below^73^:

5 $${F}_{N}\left(x\right)=\Phi \left(\frac{\text{x}-\mu }{\sigma }\right)=\frac{1}{2}\left[1+erf\left(\frac{\text{x}-\mu }{\sigma \sqrt{2}}\right)\right], -\infty <x<\infty ,$$

6 $${f}_{N}\left(x\right)=\frac{1}{\sigma \sqrt{2\pi }}{e}^{-\frac{1}{2}{ \left(\frac{x-\mu }{\sigma }\right)}^{2}}, -\infty <x<\infty ,$$

where $-\infty <\mu <\infty$ is a location and ${\sigma }^{2}>0$ is a squared scale parameter and $\Phi$ is the cdf of the standard normal distribution and $erf$ is the complementary error function.

The gamma distribution is frequently used in seismology to model earthquake inter-event times, energy release, and the duration between major seismic events. It serves as a generalization of the Poisson process by allowing for variable waiting times between events, making it particularly suitable for characterizing clustered or time-dependent seismic sequences. Unlike the exponential distribution, which assumes a constant hazard rate, the Gamma distribution accommodates rate variability through its shape parameter α\alphaα, thereby offering greater flexibility in modeling temporal dependencies and memory effects in earthquake occurrence patterns. For example, a shape parameter α > 1 corresponds to a system where the hazard rate increases with time, which is often observed in aftershock sequences or stress accumulation processes^16,74^. Additionally, the gamma distribution is capable of representing cumulative energy dissipation, as it can model skewed and heavy-tailed distributions, a characteristic commonly observed in seismic energy release patterns. In this context, it has been applied to model both the frequency of earthquake occurrences within fixed time windows and the distribution of seismic moments or inter-event durations^75,76^. The cdf and pdf of the Gamma distribution are given below

7 $${F}_{G}\left(x\right)=\frac{1}{\Gamma \left(\alpha \right)}\gamma \left(\alpha ,\beta \right), x>0,$$

8 $${f}_{G}\left(x\right)=\frac{{\beta }^{\alpha }}{\Gamma \left(\alpha \right)}{x}^{\alpha -1}{e}^{-\beta x}, x>0,$$

where $\alpha >0$ is a shape and $\beta >0$ is a rate parameter and $\gamma \left(\alpha ,\beta \right)$ is the lower incomplete gamma function.

The exponential distribution is one of the most commonly used models in seismology for representing the time intervals between successive earthquake events, particularly in memoryless processes where the occurrence of one event does not influence the likelihood of another in the near future. It is often employed to model the inter-event times in a Poisson process, where events occur independently and with a constant average rate^77,78^. This distribution is especially relevant in early-stage seismic hazard assessments or when modeling background seismicity, where earthquake occurrences are assumed to be random and temporally independent. Its main advantage lies in its simplicity and closed-form expressions for both the pdf and cdf

9 $${F}_{E}\left(x\right)=1-{e}^{-\lambda x}, x\ge 0,$$

10 $${f}_{E}\left(x\right)=\lambda {e}^{-\lambda x}, x\ge 0,$$

where $\lambda >0$ is rate or inverse scale parameter.

In the context of earthquake modeling, the exponential distribution serves as a benchmark model for more complex time-dependent models. For instance, if observed inter-event times significantly deviate from an exponential distribution, this may indicate temporal clustering, stress transfer, or aftershock triggering processes, suggesting the need for alternative models like Gamma, Weibull, or ETAS (Epidemic-Type Aftershock Sequence) models^79,80^. Although it provides a reasonable approximation for low-magnitude background seismicity, the exponential model generally underestimates the likelihood of short inter-event times during aftershock sequences and fails to account for the long-term memory or rate changes in seismic activity, which are often critical for hazard prediction. Nonetheless, due to its interpretability and mathematical tractability, the exponential distribution remains a foundational tool in probabilistic seismic hazard analysis^81^ and is widely used in parametric models to approximate recurrence intervals, triggering probabilities, and return periods in earthquake-prone regions.

The Weibull distribution is widely employed in seismic hazard analysis due to its flexibility in modeling the distribution of earthquake magnitudes, inter-event times, and energy release. It is particularly effective in reliability-based models where failure times or extreme occurrences are of interest. The distribution’s scale $(\alpha$) and shape (λ) parameters allow it to capture various behaviors of seismic events, including those with increasing or decreasing hazard rates^75,82^. The cdf and pdf of the Weibull distribution are given below

11 $${F}_{W}\left(x\right)=1-{e}^{-{\left(\frac{x}{\lambda }\right)}^{\alpha }}, x\ge 0,$$

12 $${f}_{W}\left(x\right)=\frac{\alpha }{\lambda }{\left(\frac{x}{\lambda }\right)}^{\alpha -1}{e}^{-{\left(\frac{x}{\lambda }\right)}^{\alpha }}, x\ge 0,$$

where $a>0$ is scale and $\lambda >0$ is a shape parameter.

In seismology, a shape parameter α < 1 may suggest aftershock decay, while α > 1 indicates increasing hazard, potentially modeling stress accumulation in fault zones. The Weibull distribution has been successfully used to model earthquake recurrence intervals and lifespan of seismic sequences, particularly when the assumption of constant hazard rate (as in exponential models) does not hold^83^.

The log-normal distribution is frequently used to describe earthquake magnitudes and energy release, particularly when these variables exhibit positive skewness. In this distribution, the logarithm of the variable (e.g., magnitude) follows a normal distribution, making it appropriate for modeling skewed data in seismology^30,84^. The cdf and pdf of the log-normal distribution are defined as follows:

13 $${F}_{LN}\left(x\right)=\Phi \left(\frac{\text{ln}\left(x\right)-\mu }{\sigma }\right)=\frac{1}{2}\left[1+erf\left(\frac{\text{ln}\left(x\right)-\mu }{\sigma \sqrt{2}}\right)\right],$$

14 $${f}_{LN}\left(x\right)=\frac{1}{x\sigma \sqrt{2\pi }}{e}^{\left(- \frac{{\left(\text{ln}\left(x\right)-\mu \right)}^{2}}{2{\sigma }^{2}}\right)},$$

where $-\infty <\mu <\infty$ and $\sigma >0$ are the mean and standard deviation of the logarithm of the distribution, respectively and $\Phi$ is the cdf of the standard normal distribution and $erf$ is the complementary error function.

The Gumbel distribution, one of the primary families in extreme value theory (EVT), is particularly suitable for modeling maximum (or minimum) values of a dataset. In seismology, it is frequently used to analyze extreme earthquake magnitudes, particularly for estimating return periods and the probability of rare but catastrophic events^41,85^. Its widespread use in seismic hazard analysis stems from several theoretical and empirical advantages. The Gumbel distribution arises as the limiting distribution of the maximum of a large number of independent identically distributed (i.i.d.) random variables. This fits the context of modeling the largest magnitudes observed over certain time periods or regions. The cdf and pdf of the Gumbel distribution are, respectively, given by.

15 $${F}_{G}\left(x\right)={{e}^{-e}}^{-\left(\frac{x-\alpha }{\beta }\right)}, -\infty <x<\infty$$

16 $${f}_{G}\left(x\right)=\frac{1}{\beta }{e}^{-\left(\frac{x-\alpha }{\beta }+{e}^{- \left(\frac{x-\alpha }{\beta }\right)}\right)}, -\infty <x<\infty ,$$

where $\alpha >0$ is a location and $\beta >0$ is a scale parameter.

Earthquake magnitudes exhibit a positive skew with no upper bound (theoretically unbounded). Gumbel’s right-skewed nature and unbounded support match this empirical behavior well. The location parameter α approximates the central tendency of extremes (e.g., the threshold magnitude), and the scale parameter β captures the variability, allowing straightforward interpretation for risk communication.

### LSTM method

Although RNNs are effective in addressing long-term dependency issues, they may encounter challenges such as gradient vanishing in certain applications^86^. To overcome this limitation, Hochreiter and Schmidhuber^42^ introduced the Long Short-Term Memory (LSTM) model, a specialized type of RNN. Since its development, various researchers—such as Rodriguez et al.^87^, Medsker and Jain^88^, Hermans and Schrauwen^89^, and Graves^90^—have proposed enhancements to improve its performance. As a result, LSTM has become a widely adopted model in time series forecasting. By incorporating memory cells and gating mechanisms to control information flow, LSTM effectively addresses the gradient vanishing problem and outperforms classical RNNs^91^. Furthermore, LSTM provides strong performance in time series applications by overcoming limitations of traditional forecasting methods^92^.

**Fig. 6:**
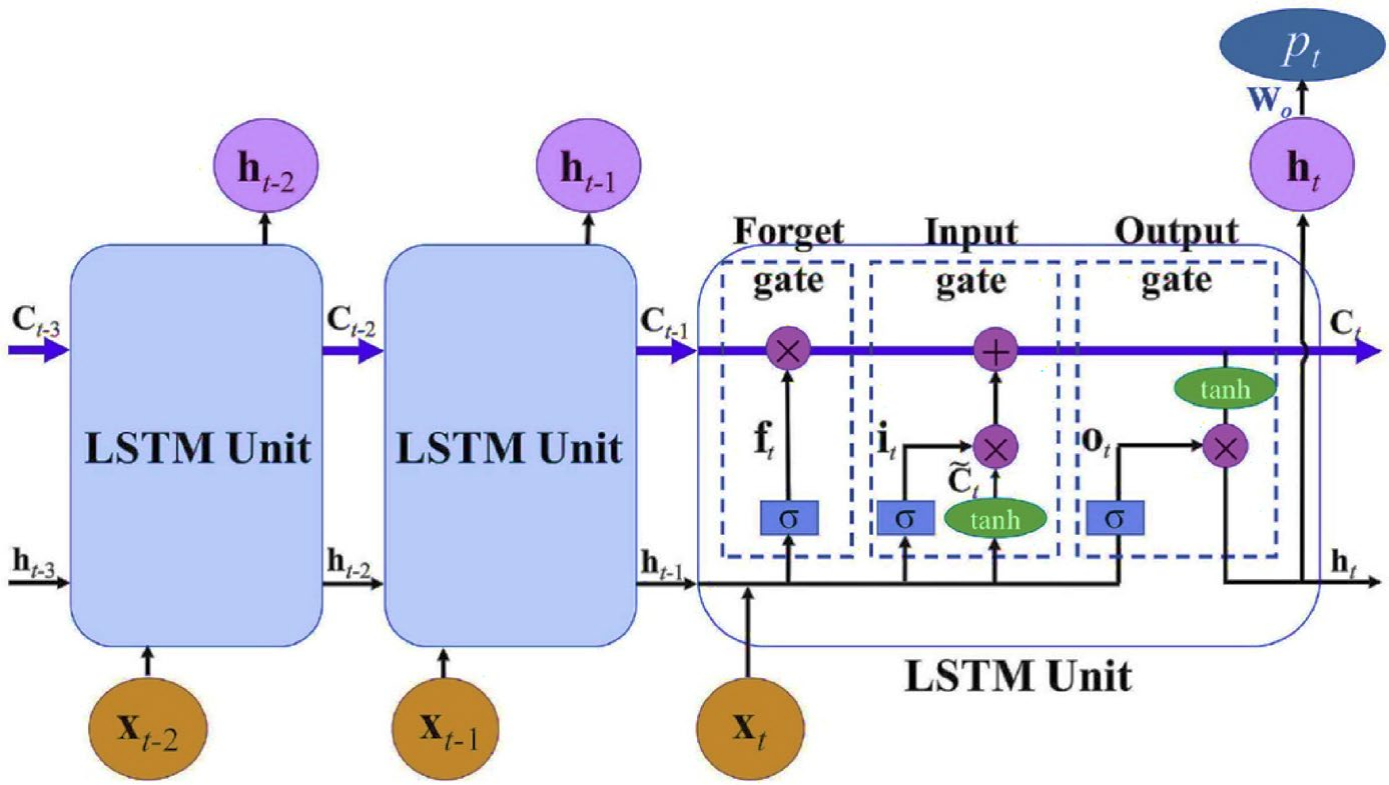
Schematic representation of a basic LSTM architecture^96^.

LSTM units consist of three main components: the input gate, forget gate, and output gate. The cell state plays a crucial role in carrying information through the network^22^. The input gate determines whether new information should be incorporated based on its relevance. The forget gate enhances learning by deciding which information should be retained or discarded. The output gate structures and transmits the processed information^93–95^. LSTM has proven to be an effective algorithm for time series modeling, and its operational structure is illustrated in Fig. 6.

The input gate is used to define what new information should be recorded in the cell state. It has two main components, namely, the sigmoid layer and *tanh* layer. The sigmoid determines what information to record in the cell state, while *tanh* generates a vector of new candidate values to place in the cell state. The outputs of the sigmoid layer and *tanh* layer are computed in the following way:

17 $${i}_{t}=\sigma \left({W}_{{i}_{h}}\left[{h}_{t-1}\right]+{W}_{{i}_{x}}\left[{X}_{t}\right]+{b}_{i}\right),$$

18 $$\widetilde{{c}_{t}}=tanh\left({W}_{{c}_{h}}\left[{h}_{t-1}\right]+{W}_{{c}_{x}}\left[{X}_{t}\right]+{b}_{c}\right).$$

New information to be added to the cell state is calculated by combining the outputs of the sigmoid layer and *tanh* layer:

19 $${c}_{t}={f}_{t}\odot {c}_{t-1}+{i}_{t}*\widetilde{{c}_{t}}.$$

The forget gate output is then used in the element-wise multiplication represented by the symbol ⊙. The forget gate output, ${f}_{t}$, determines the data to be extracted from the cell state. It is calculated using ${X}_{t}$ and ${h}_{t-1}$, and a sigmoid layer. The output of the forget gate is a value between 0 and 1, where 0 means that the data could be completely forgotten and 1 means that it should be retained.

20 $${f}_{t}=\sigma \left({W}_{{f}_{h}}\left[{h}_{t-1}\right]+{W}_{{f}_{x}}\left[{X}_{t}\right]+{b}_{f}\right).$$

The forget gate ${f}_{t}$ output combines the previous memory cell signal with the signal ${c}_{t-1}$. The resulting element-wise multiplication (⊙) with ${c}_{t-1}$ determines which data to keep and which to forget:

21 $${c}_{t}={f}_{t}\odot {c}_{t-1}.$$

The output gate determines which data is output. It makes a major contribution to the selection of the part of the LSTM memory that will be effective. It uses a sigmoid layer followed by a *tanh* layer, mimicking the process of the input gate. The sigmoid layer calculates the importance and is multiplied element-wise by its output using a non-linear *tanh* function. The process is obtained by

22 $${o}_{t}=\sigma \left({W}_{{o}_{h}}\left[{h}_{t-1}\right]+{W}_{{o}_{x}}\left[{X}_{t}\right]+{b}_{o}\right),$$

23 $${h}_{t}={o}_{t}\odot \text{tanh}\left({c}_{t}\right).$$

The vector of candidate cell values $\widetilde{{c}_{t}}$ is given by and the vector of the memory cell by ${c}_{t}$. Furthermore, the sigmoid function $\sigma$ and the output matrix ${h}_{t}$ are defined. Weight matrices ${W}_{i}$, ${W}_{f}$, ${W}_{o}$ and ${W}_{c}$, along with bias vectors ${b}_{i}$, ${b}_{f}$, ${b}_{o}$ and ${b}_{c}$ are used. The result output ${h}_{t}$ is the element-wise product of ${o}_{t}$ and the hyperbolic tangent of the current memory cell state ${c}_{t}$. This result carries the data to form the prediction in the LSTM process. The sources cited for these informations are Reyes et al.^97^, Gürsoy et al.^98^ and Jia and Ye^99^.

## Results and discussion

### Clustering with FCM of earthquake catalog

In this section, we first present the basic characteristics of the earthquake catalog prior to conducting the clustering analysis using the FCM algorithm. Although the complete catalog includes seismic events recorded between 1900 and 2021, the majority of events documented in the KOERI and Tan^51^ catalog occurred after 1970. Given the lack of sufficient and reliable data before this date, the analyses in this study are limited to the period from January 1970 to September 2021. Table 1 presents the key descriptive statistics for earthquake magnitudes and depths over this selected timeframe. For depth, the first quartile (Q_1_) is 6.81 km, the median (Q_2_) is 10 km, and the third quartile (Q_3_) is 16.32 km. The average depth is 16.06 km, with a standard deviation of 21.25 km. The skewness of 3.84 and kurtosis of 20.79 indicate a highly right-skewed and heavy-tailed distribution. Regarding earthquake magnitudes during the same period, Q_1_ is 2.54, the median is 2.76, and Q_3_ is 3.20. The mean magnitude is 2.96, with a standard deviation of 0.60. The skewness (1.54) and kurtosis (5.38) values again suggest a right-skewed distribution with heavier tails than a normal distribution.

**Table 1:** Basic statistics for earthquake magnitude and depth (01.1970–09.2021).

	Depth (km)	Magnitude (Mwg)		Depth (km)	Magnitude (Mwg)
Minimum	0	2.43	Maximum	183.80	7.50
Q_1_	6.81	2.54	St. Deviation	21.25	0.60
Q_2_	10.00	2.76	Skewness	3.84	1.54
Mean	16.06	2.96	Kurtosis	20.79	5.38
Q_3_	16.32	3.20			

**Fig. 7:**
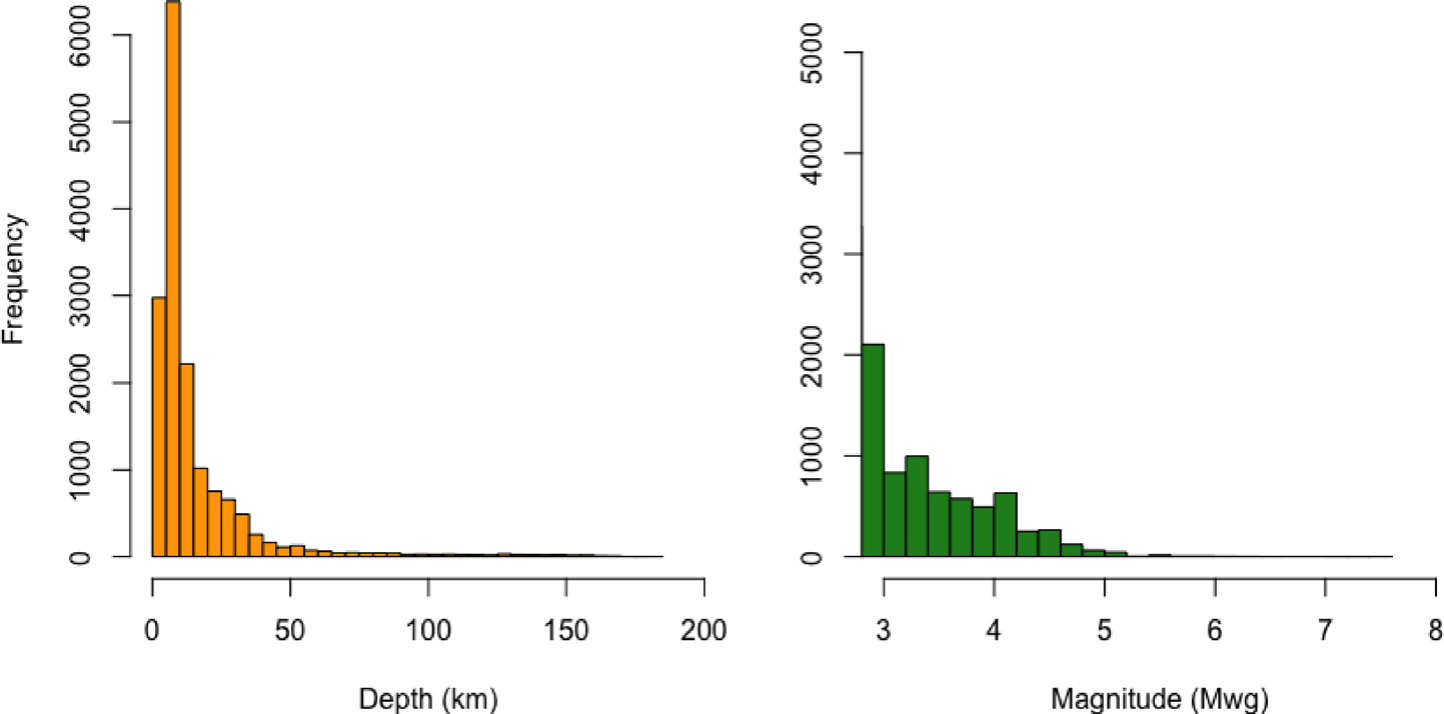
Frequency distributions of earthquake depth and magnitude (Mwg) recorded between 1970 and 2021.

Figure 7 presents the histograms of earthquake depth and magnitude for the period between 1970 and 2021. Both distributions are positively skewed, indicating that most earthquakes occurred at shallow depths and had relatively low magnitudes. The depth histogram (left) shows that the majority of earthquakes occurred at depths less than 20 km, with a sharp decline in frequency as depth increases. Similarly, the magnitude histogram (right) reveals that most events were of low to moderate magnitude, predominantly clustered around 3.0–4.0 Mwg.

**Fig. 8:**
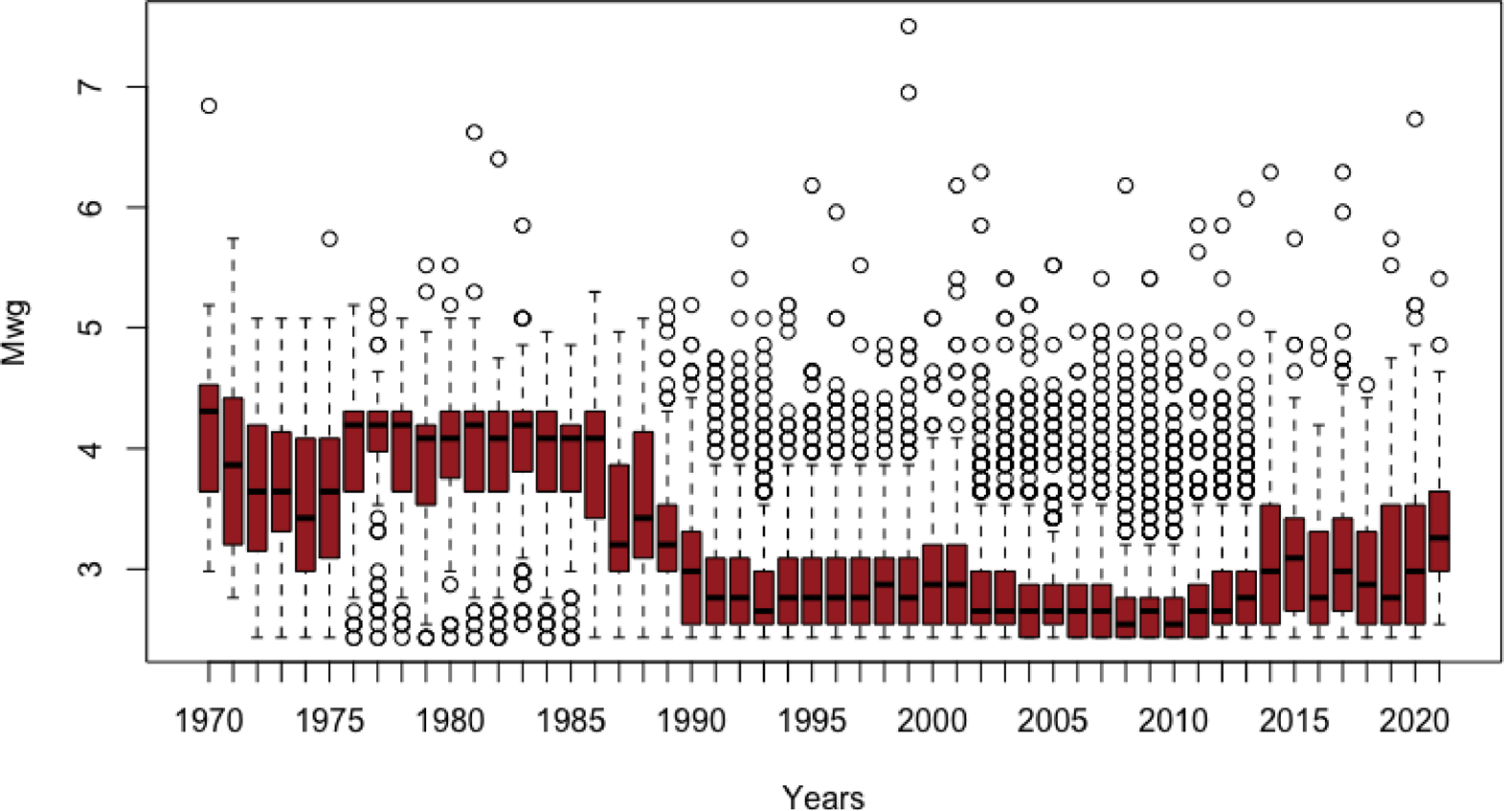
Temporal distribution of earthquake magnitudes (Mwg) from 1970 to 2021 based on yearly box plots.

The box plots in Fig. 8 illustrate the yearly distribution of earthquake magnitudes (Mwg) recorded between 1970 and 2021. A noticeable downward trend in magnitudes is observed after 1990. This apparent decline is likely attributable to the increased sensitivity and improved resolution of earthquake recording instruments introduced in the early 1990s, which allowed for more frequent detection of lower-magnitude events.

**Fig. 9:**
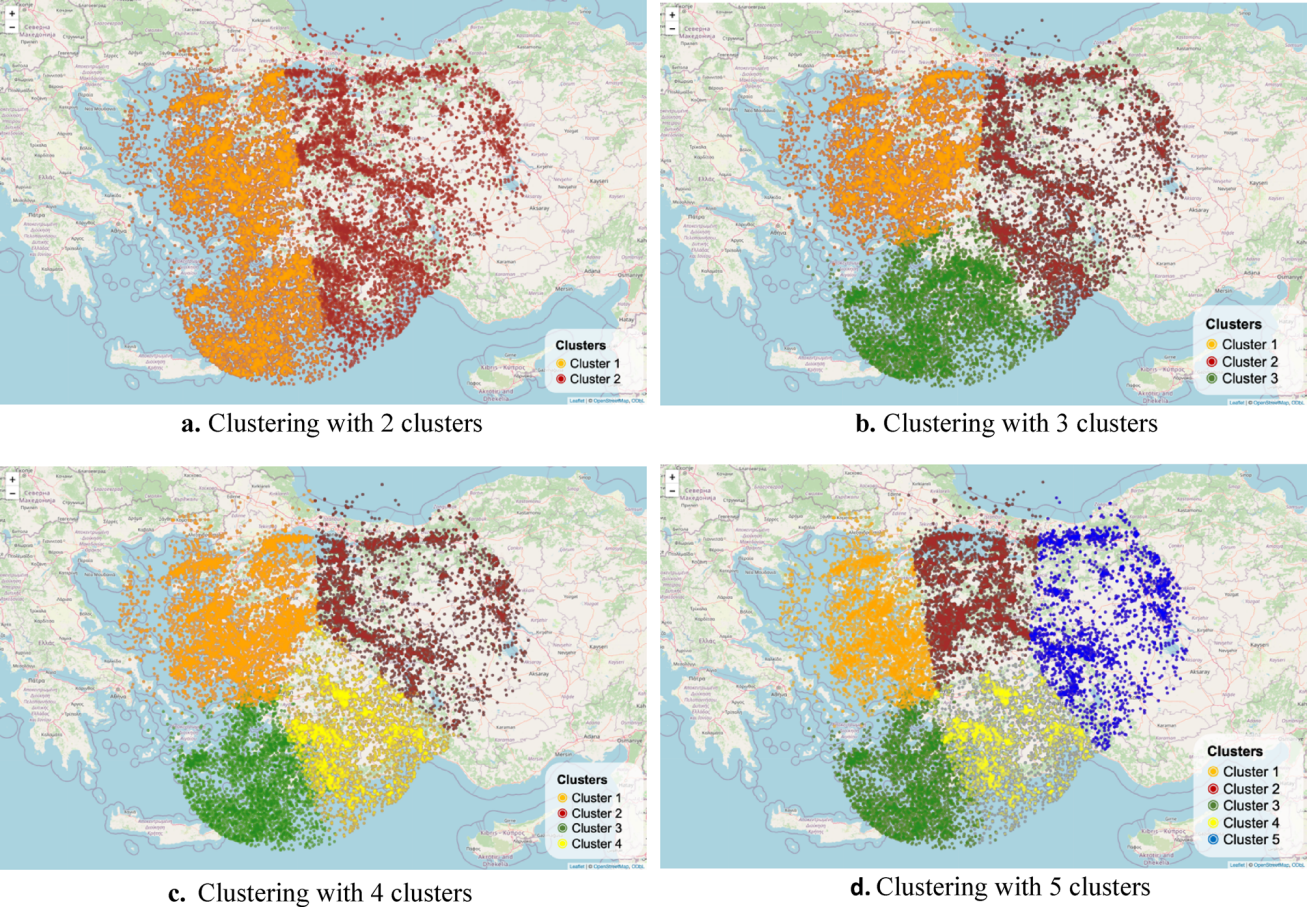
Spatial clustering results of the declustered earthquake catalog using the FCM algorithm (R version 4.3.1; https://www.r-project.org/).

The FCM algorithm was employed to partition the earthquakes into homogeneous subgroups. The analysis was based on the latitude, longitude, and magnitude values of earthquakes occurring within the defined study area. The FCM algorithm was applied for varying numbers of clusters, specifically from 2 to 5, to evaluate different levels of granularity in spatial segmentation. The resulting cluster structures for each case are illustrated in Fig. 9a–d.

**Fig. 10:**
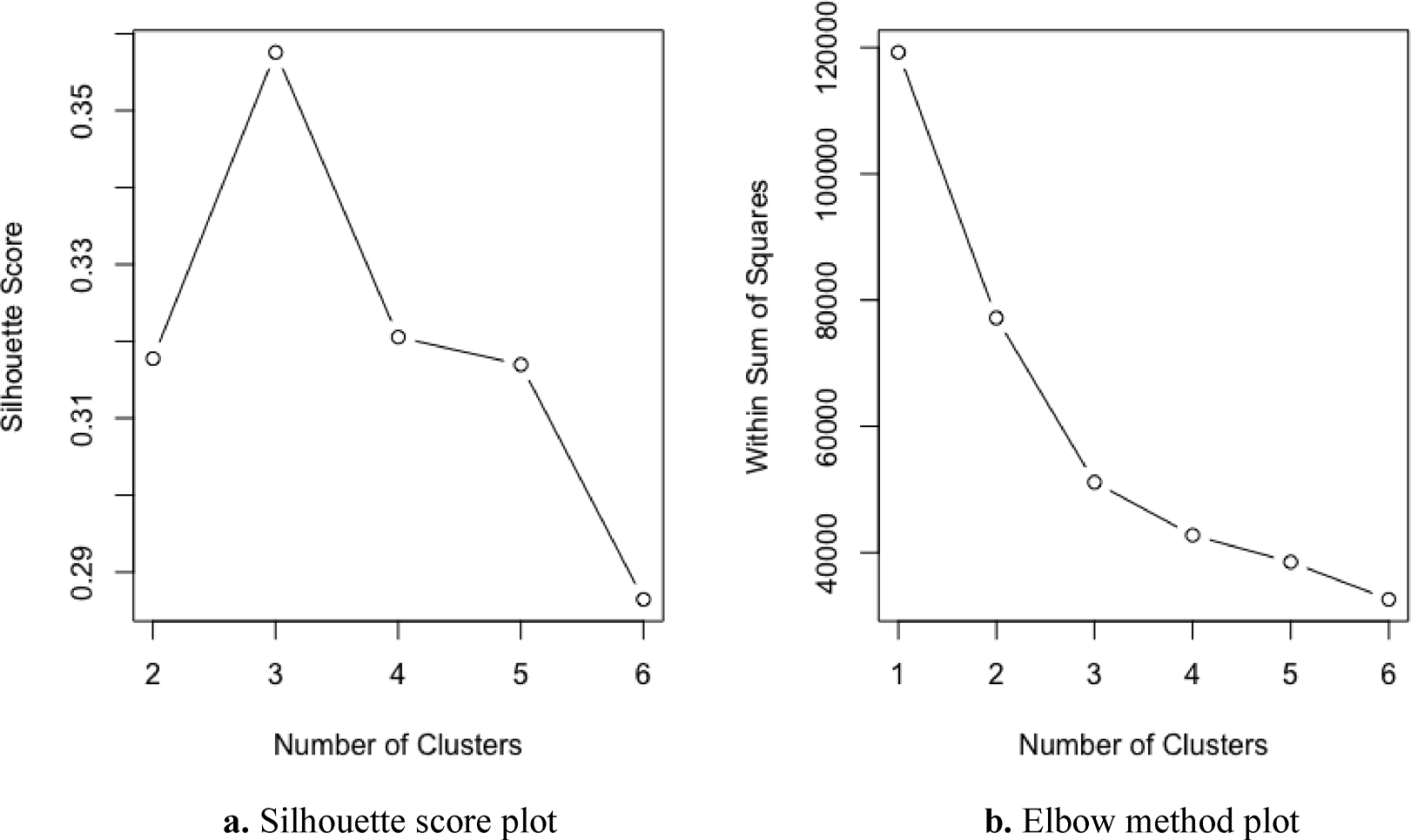
Silhouette score (**a**) and Elbow method (**b**) plots for determining the optimal number of clusters in the FCM analysis of earthquake data (1970–2021).

The clustering results shown in Fig. 9 are based on the FCM algorithm, where each event has a degree of membership in multiple clusters. For visualization purposes, each event is assigned to the cluster in which it has the highest membership value. Unlike deterministic clustering methods, FCM does not create sharp boundaries, as events near the cluster edges exhibit overlapping characteristics. The cluster regions should, therefore, be interpreted as areas of influence rather than strictly separated zones. Figure 10 displays the elbow criterion and the silhouette score results for the FCM analysis. These graphs are crucial in determining the appropriate cluster number in clustering analysis. The Silhouette Score graph illustrates the clustering quality for different numbers of clusters and the Elbow Criterion graph shows the explained variance versus the number of clusters.

According to both the silhouette score and the Elbow method, the optimal number of clusters for the FCM analysis is determined to be three. The silhouette plot indicates a peak at *K* = 3, suggesting well-defined and compact clusters, while the Elbow plot shows a clear inflection point at the same value. Figure 10b visually supports this conclusion by illustrating the sharp decrease in within-cluster variance up to *K* = 3, after which the marginal gain diminishes. Accordingly, the clustering results for *K* = 3 are further analyzed and presented in the following figures, and the details of each cluster are summarized in Table 2.

**Table 2:** Summary statistics of earthquake clusters identified by the FCM algorithm based on magnitude and spatial coordinates (1970–2021).

Cluster	Magnitude (Mwg) mean	Longitude mean	Latitude mean	Number of incidents	Magnitude (Mwg) variance
1	2.89	26.73	39.32	5813	0.2997
2	2.85	30.51	38.87	4682	0.3076
3	3.15	27.63	36.33	5350	0.4130

**Fig. 11:**
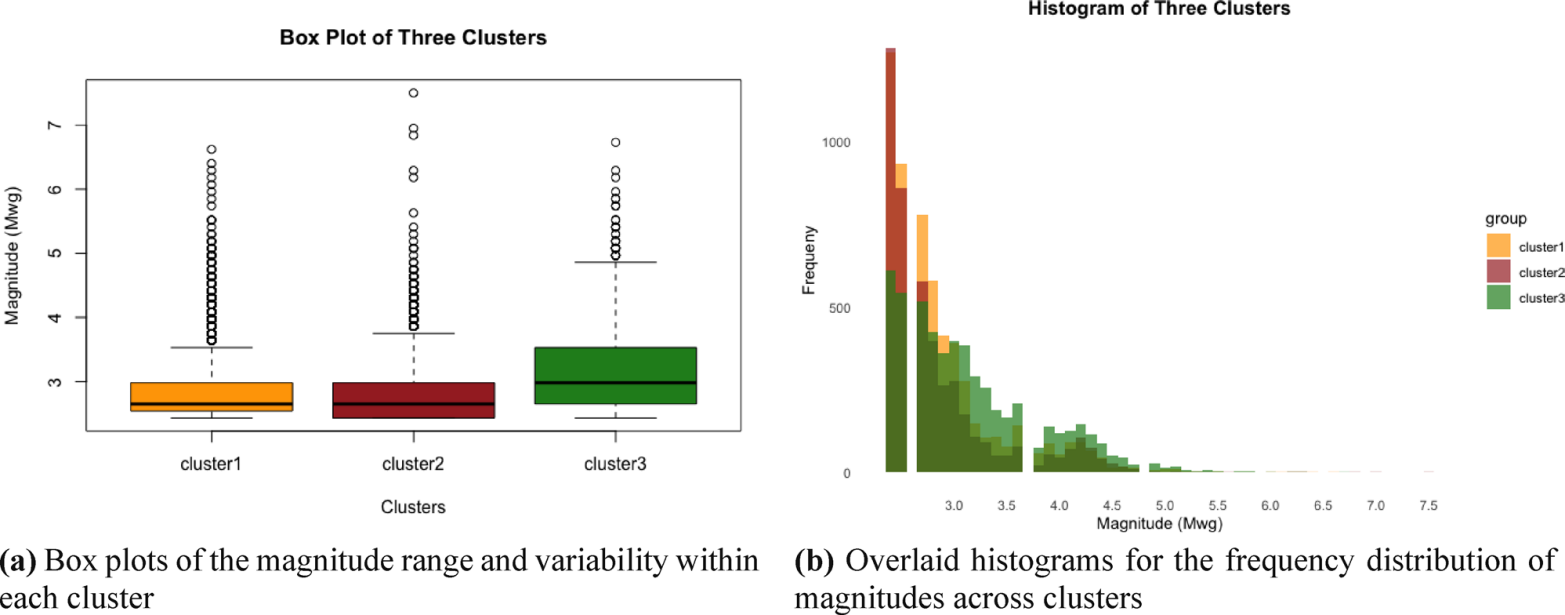
Magnitude distributions of the three clusters identified by the FCM algorithm (1970–2021).

In Table 2, the earthquake catalogue is divided into three clusters, each with different characteristics. The first cluster, which contains the largest number of earthquakes (5813), includes earthquakes with depths of up to 164 km and magnitudes between 2.43 Mwg and 6.62 Mwg. The average magnitude of the earthquakes occurring in this cluster is 2.89 Mwg. The second cluster, consisting of 4682 earthquakes, has a similar average magnitude of 2.85 Mwg. This cluster mainly includes events occurring on land, with earthquake depths reaching 159.2 km. The third cluster, containing 5350 earthquakes, is concentrated in offshore areas, with an average magnitude of 3.15 Mwg and a maximum depth of 183.8 km. In particular, the devastating Izmir earthquake of October 30, 2020 is part of this cluster. Cluster 1 stands out for having the largest membership compared to the others, while Cluster 2, despite having the fewest observations, contains some of the strongest earthquakes in the region. The box plot and histogram graphs for the clusters are, respectively, presented in Fig. 11a,b. From these graphs, it can be observed that Cluster 3 is slightly different from the other clusters.

**Fig. 12:**
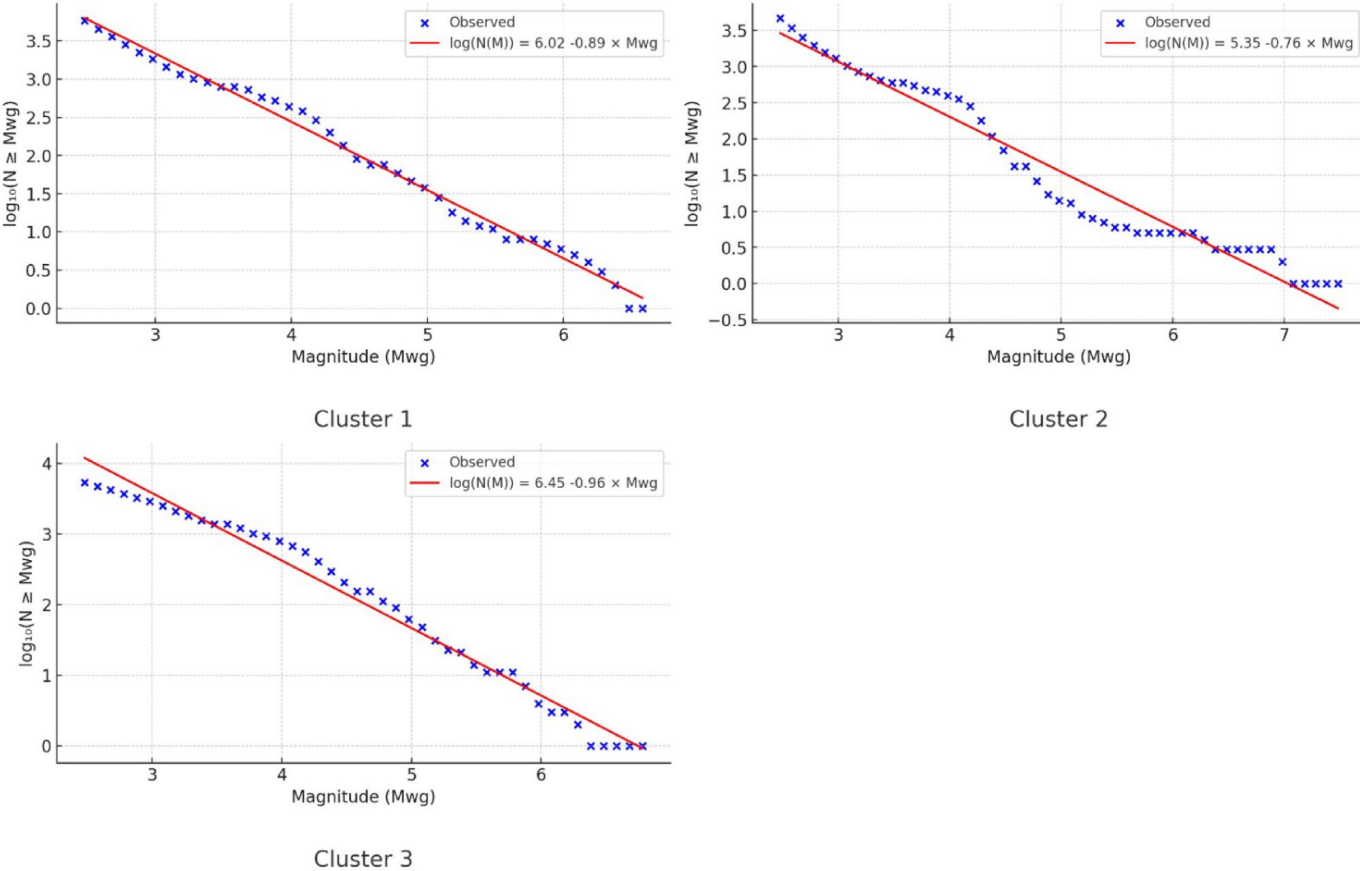
Gutenberg-Richter plots for each cluster.

The seismicity of seismic sources is also assessed using the Gutenberg-Richter relation. It is obtained as $Log N\left(M\right)=6.80-0.99M$ for the first cluster, $Log N\left(M\right)=6.01-0.84M$ for the second cluster and $Log N\left(M\right)=7.31-1.07M$ for the third cluster, as illustrated in Fig. 12, respectively. The Gutenberg–Richter plots indicate distinct seismic characteristics for each cluster. Cluster 1 exhibits moderate seismic activity with a typical *b*-value close to 1, suggesting a balanced occurrence of small and large earthquakes. Cluster 2 shows the lowest seismic activity, as indicated by its relatively low *a*-value; however, its *b*-value below 1 suggests that larger earthquakes are more likely in this region. In contrast, Cluster 3 demonstrates the highest seismic activity (highest a-value), and a *b*-value greater than 1, implying that small-to-moderate magnitude events dominate over large ones.

### Statistical modeling of magnitude distributions

In this section, the D’Agostino skewness test is applied to evaluate the asymmetry in the distribution of earthquake magnitudes for each cluster, while the Anscombe–Glynn kurtosis test is employed to assess tail behavior. The results of these statistical tests, along with descriptive measures for magnitudes, are presented in Table 3.

**Table 3:** Descriptive statistics and normality test results of Mwg values for each cluster.

	Min	Q_1_	Median	Mean	Q_3_	Max	St. Dev
Cluster 1	2.4	2.5	2.6	3.4	3.5	6.6	0.5
Cluster 2	2.4	2.4	2.7	2.8	3.0	7.5	0.6
Cluster 3	2.4	2.7	3.0	3.1	3.5	6.7	0.6

	Skewness			Kurtosis			
	Value	Z-value	*P*-value	Value	Z-value	*P*-value	
Cluster 1	1.85	39.59	0.0	6.8	22.67	0.0	
Cluster 2	2.0	37.21	0.0	7.73	22.32	0.0	
Cluster 3	1.07	26.59	0.0	3.81	8.78	0.0	

Table 3 summarizes the descriptive statistics and normality test results for earthquake magnitudes (Mwg) across the three clusters. In Cluster 1, the magnitude distribution has a mean of 3.4 and median of 2.6, with a moderate spread (St. Dev. = 0.5). The distribution is positively skewed (Skewness = 1.85; Z = 39.59; *P* = 0.000) and exhibits high kurtosis (6.8; Z = 22.67; *P* = 0.000), indicating a significant deviation from normality due to the presence of heavy tails and asymmetry. In Cluster 2, the magnitudes exhibit slightly less skew (Skewness = 2.00) but a similar pattern of deviation (Kurtosis = 7.73). The variability is highest here (St. Dev. = 0.6), and the maximum observed value reaches 7.5. The normality test again reveals a statistically significant deviation from normality (*P* < 0.001), confirming the influence of extreme values. Cluster 3 displays a more concentrated magnitude range with a mean of 3.1, median of 3.0, and standard deviation of 0.6. Although the skewness is lower (1.07), the kurtosis is 3.81, and the normality test still yields significant results (Z = 8.78; *P* = 0.000), implying a sharper peak and non-normal characteristics.

**Fig. 13:**
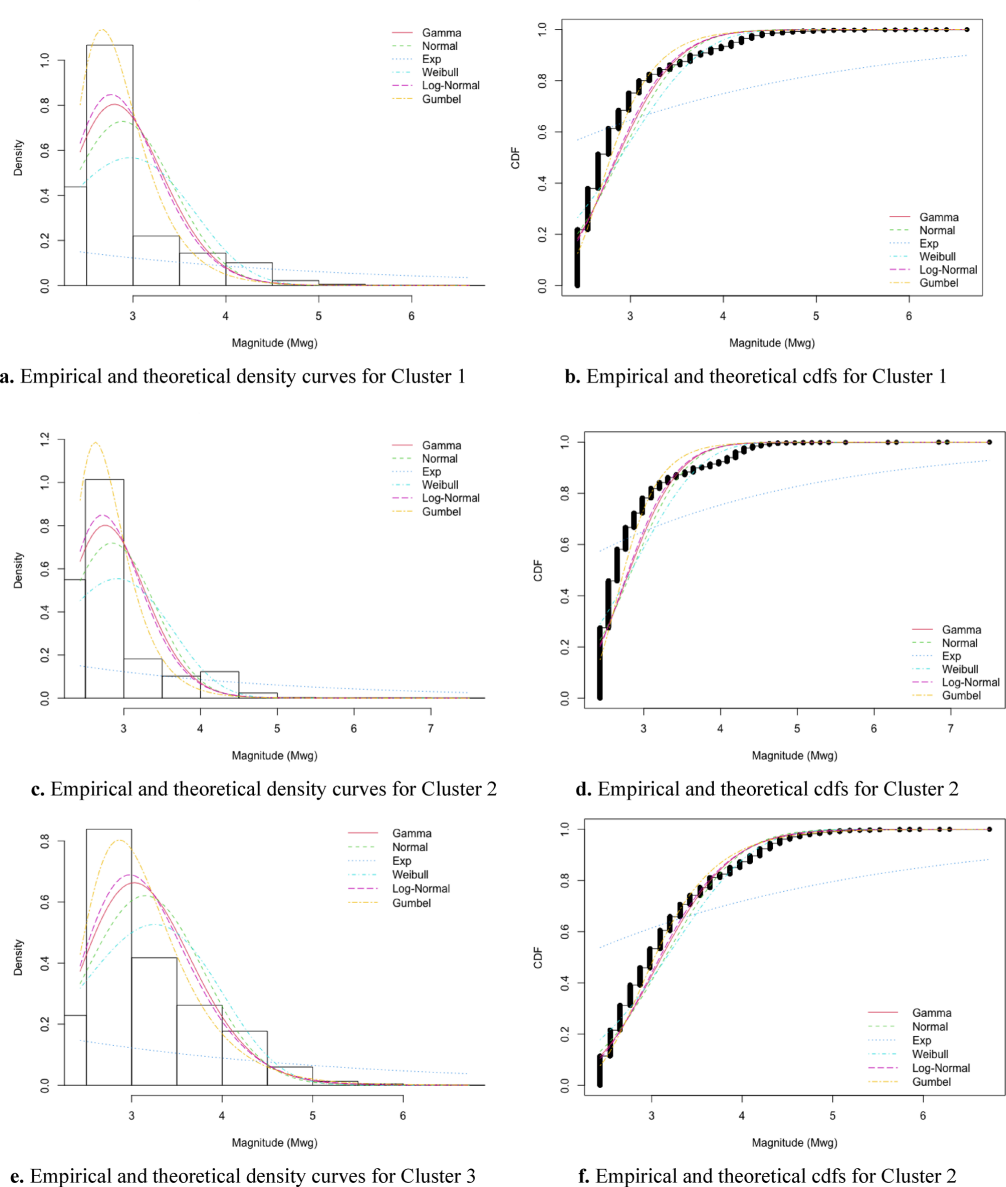
Histograms and plots of empirical and theoretical density and distribution functions for each cluster.

Figure 13e,f suggest that the Gumbel distribution provides a relatively better fit for Cluster 3. However, for Clusters 1 and 2, a definitive conclusion regarding the best-fitting distribution could not be drawn based solely on visual inspection of Fig. 13a–d. To complement the graphical analysis, two formal goodness-of-fit tests Kolmogorov–Smirnov (K–S) and Cramér–von Mises (CVM) were conducted. The results of these tests are presented in Table 4. The K–S test measures the maximum deviation between the empirical cumulative distribution function (cdf) and the theoretical cdf. The CVM test, similar in purpose, places greater emphasis on discrepancies throughout the entire distribution, especially in the tails.

**Table 4:** Summary of goodness-of-fit test results for each cluster and distribution.

	Gamma	Normal	Exponential	Weibull	Log-Normal	Gumbel
Cluster 1						
K-S	0.19131	0.20485	0.56898	0.26546	0.18345	0.16507
CVM	57.51791	72.09748	414.80405	88.07294	51.04678	31.18292
Cluster 2						
K-S	0.11724	0.14008	0.53782	0.17829	0.11365	0.10845
CVM	18.97300	26.96697	347.54976	32.73997	15.87689	10.80354
Cluster 3						
K-S	0.2140693	0.227944	0.574199	0.2898438	0.20934	0.1944553
CVM	62.4569180	74.833173	336.632628	86.9155562	56.41904	37.8755302

To evaluate the performance of the fitted distributions and support model selection, we employ two widely used information criteria: the Akaike Information Criterion (AIC) and the Bayesian Information Criterion (BIC). The AIC is based on maximum likelihood estimation and offers a trade-off between model fit and complexity. It is calculated using the formula AIC = –2 × log-likelihood + 2*p*, where *p* represents the number of parameters. Lower AIC values indicate a better-fitting model by penalizing over-parameterization. The BIC, derived from Bayesian principles, introduces a stronger penalty for model complexity and is computed as BIC = –2 × log-likelihood + log(*n*) × *p*, where *n* is the sample size. As with AIC, lower BIC values suggest a more suitable model. Table 5 presents the AIC and BIC values for each distribution fitted to the three clusters. According to the results, the Gumbel distribution consistently yields the lowest AIC and BIC values across all clusters, indicating the best overall fit. The corresponding maximum likelihood estimates (MLEs) of the distribution parameters are provided in Table 6.

**Table 5:** Comparison of distributional fit using AIC and BIC across clusters.

	Gamma	Normal	Exponential	Weibull	Log-Normal	Gumbel
Cluster 1						
AIC	8381.640	9496.515	23,956.960	10,885.320	7899.653	6402.509
BIC	8394.976	9509.851	23,963.630	10,898.660	7912.989	6415.845
Cluster 2						
AIC	6782.004	7770.779	19,161.590	8916.539	6349.167	4948.955
BIC	6794.907	7783.682	19,168.044	8929.442	6362.070	4961.858
Cluster 3						
AIC	9791.482	10,455.990	22,975.390	11,196.460	9534.664	9010.250
BIC	9804.652	10,469.160	22,981.970	11,209.630	9547.834	9023.420

**Table 6:** Estimated parameters and standard errors for the Gumbel model by cluster.

Cluster	Parameter	Estimate	Std. Error
1	a	2.668174	0.00440
	b	0.323523	0.00369
2	a	2.629643	0.00468
	b	0.309822	0.004012
3	a	2.863809	0.006561
	b	0.457912	0.005215

The suitability of the Gumbel distribution for modeling earthquake magnitudes in this study is supported by both seismological considerations and theoretical properties of extreme value modeling. Earthquake magnitudes are inherently right-skewed, bounded below by a catalog completeness threshold (e.g., Mwg ≥ 2.43), and unbounded above, with the primary interest often focused on large, destructive events. The Gumbel distribution, a member of the EVT family, is specifically designed to model the statistical behavior of maximum values and thus offers a natural framework for capturing the tail behavior of seismic data^41^. In the context of the Aegean region, the magnitude data exhibit asymmetry and heavy-tailed behavior. This structure aligns well with the shape characteristics of the Gumbel distribution, which provides flexibility in modeling rare and extreme events without imposing an artificial upper bound. Previous studies have shown that Gumbel-based models perform reliably in modeling maximum yearly magnitudes, extreme aftershocks, and peak ground motion parameters^83,85^. In addition to its theoretical appropriateness, the Gumbel distribution outperformed alternative models such as the Normal, Log-Normal, Weibull, and Gamma distributions in this study based on goodness-of-fit criteria (K–S, CVM, and AD tests) and information criteria (AIC and BIC). These results reinforce its validity as a robust tool for representing the extreme behavior of regional earthquake magnitudes.

**Fig. 14:**
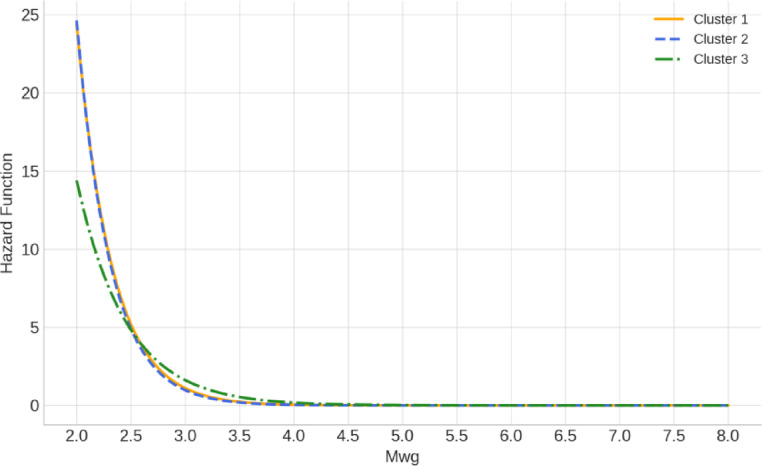
Hazard function curves derived from the Gumbel model for each earthquake cluster.

Figure 14 illustrates the hazard functions for earthquake magnitudes across the three clusters. In reliability and survival analysis, the hazard function, also known as the failure rate or instantaneous event rate, is defined as the conditional probability that an event occurs in an infinitesimally small interval, given that it has not occurred before that time. In the context of this study, the hazard function is interpreted as the instantaneous probability of an earthquake of a given magnitude occurring, given that it has not yet occurred. The hazard function is given by

$$h\left(x\right)=\frac{f\left(x\right)}{1-\text{F}\left(x\right)}=\frac{\frac{1}{\beta }exp\left(-\frac{x-\mu }{\beta }\right)exp\left(-exp\left(-\frac{x-\mu }{\beta }\right)\right)}{1-exp\left(-exp\left(-\frac{x-\mu }{\beta }\right)\right)},$$

where $f\left(x\right)$ and $\text{F}\left(x\right)$ are the pdf and cdf of the fitted Gumbel distribution, and $\alpha >0$ and $\beta >0$ are location and scale parameters, respectively^108^.

This approach has been used in various earthquake hazard and extreme value modeling studies to better understand the likelihood of occurrence of large-magnitude events^41,83^. As seen in Fig. 14, while Cluster 3 exhibits the steepest rise in the hazard function, indicating a concentrated risk within a narrower magnitude range, Cluster 2 shows a broader hazard profile, consistent with its potential for large-magnitude earthquakes due to its tectonic setting. Cluster 1 falls in between, with a moderate hazard function. These differences reflect the varying seismic characteristics of each cluster. Although Cluster 2 exhibits a lower hazard curve than Cluster 3, its lower b-value (Fig. 12) and tectonic setting suggest a higher likelihood of large-magnitude earthquakes, consistent with subduction zone dynamics.

The return periods of earthquakes with Mwg ≥ 5.5 using the Gumbel model are presented for each cluster in Table 7. As seen from the table, the return periods in Cluster 1 increase from 2.56 years at the 25% probability level to 3.63 years at the 95% level. In Cluster 2, the return periods follow a similar trend, ranging from 2.53 to 3.55 years, reflecting slightly shorter recurrence intervals compared to Cluster 1. Cluster 3, on the other hand, exhibits noticeably longer return periods across all probability levels, with values ranging from 2.71 to 4.22 years, suggesting less frequent occurrence of earthquakes above magnitude 5.5 within this group.

**Table 7:** Estimated return periods (in years) for earthquakes with Mwg ≥ 5.5 based on the Gumbel distribution model for each cluster at selected probability levels.

Probability level	Return Period (Year)		
	Cluster 1	Cluster 2	Cluster 3
%25	2.56	2.53	2.71
%50	2.79	2.74	3.03
%75	3.07	3.02	3.43
%95	3.63	3.55	4.22

### Magnitude prediction results with LSTM algorithm

After the clusters were obtained by FCM method, the LSTM analysis was used for each cluster to predict the monthly maximum magnitudes along the Aegean Region. The data was split into 70% for training and 30% for testing, with Min–Max normalization applied for consistency^100^. The LSTM model architecture was defined with the *tanh* activation function and "*return_sequences* = *True*" to maintain output sequences. Stochastic gradient descent was used for optimization, with root mean squared error (RMSE) as the loss function and k-fold cross-validation was employed to assess the model’s generalization performance. After training, the model was used for predictions, providing insights into potential monthly maximum earthquake magnitudes for each cluster. Other hyper parameters for the regions are shown in Table 8.

**Table 8:** Hyper parameter settings and RMSE values of the LSTM model for each earthquake cluster.

Clusters	Cluster 1	Cluster 2	Cluster 3
Batch size	16	128	16
Epoch number	750	1000	750
Learning Rate	0.001	0.001	0.001
Dropout Rate	0.2	0.2	0.2
RMSE (Train)	0.07	0.09	0.06
RMSE (Test)	0.08	0.11	0.08

**Fig. 15:**
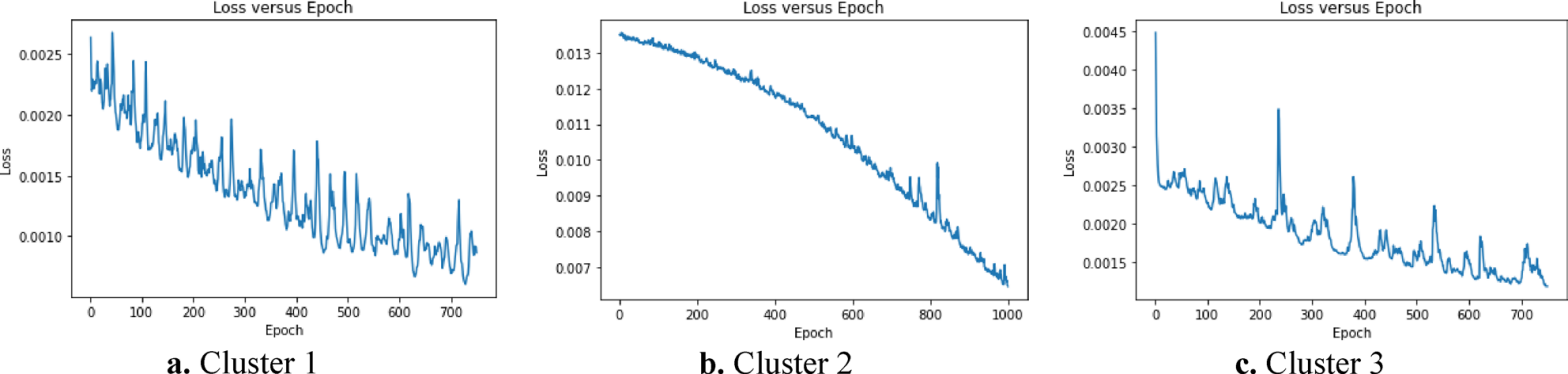
Loss values across training epochs for each cluster during LSTM model training.

Figure 15 presents the iteration number and the associated loss function values for the clusters which show the error amounts of the clusters. When the number of iterations and loss function values of the clusters are compared in Fig. 15, it is not found any significant difference between them.

**Fig. 16:**
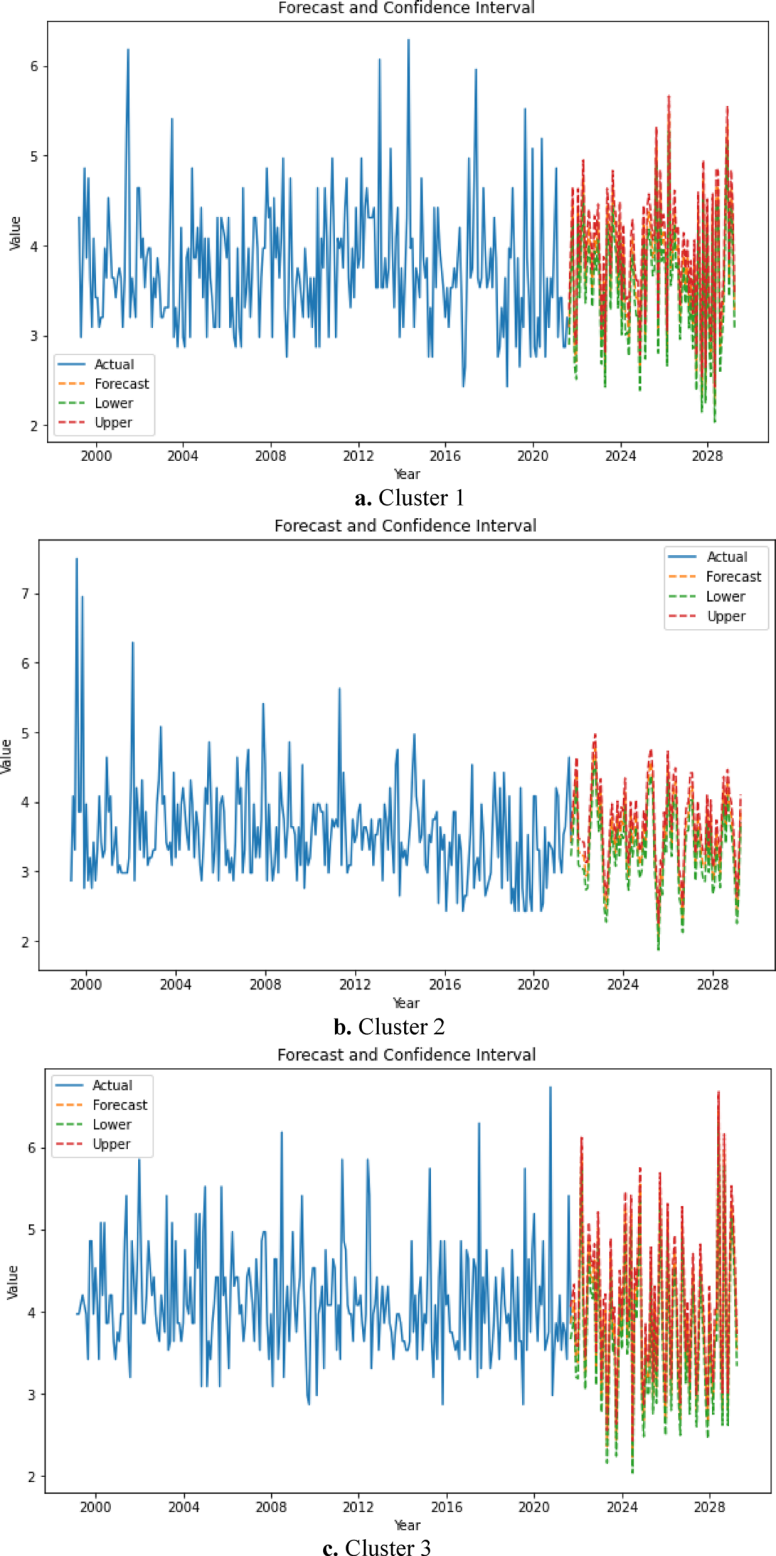
The forecast and confidence level values of magnitudes of each cluster for the period 01.2021–03.2029.

Figure 16 shows the forecasted values and confidence limits for the maximum earthquake magnitudes until March 2029. Figure 16 indicates that the maximum earthquake magnitudes in the upcoming years closely align with the maximums in the 1st (the region between the cities of Istanbul, Uşak, Athens and Thessaloniki) and 2nd (the region between the cities of Istanbul, Zonguldak, Aksaray and Antalya) clusters. It should be emphasized that the maximum magnitudes within the 3rd cluster (the region between the cities of Izmir, Athens, Crete and Antalya) exhibit a tendency toward higher values.

When examining the forecasts from October 2021 to March 2029, the highest maximum predicted magnitudes obtained for the 1^st^, 2^nd^ and 3^rd^ clusters are 5.7, 5.0, and 6.5, respectively. This implies that the LSTM-generated prediction model anticipates no earthquakes exceeding a monthly maximum magnitude of 5.7 for the first and second clusters over the period 10.2021–03.2029. However, for the third cluster, it is forecasted that earthquakes of a monthly maximum magnitude of 6.5 may occur in the year 2028. The region in the third cluster includes major metropolitan areas such as Izmir, Muğla, Athens, Crete, Antalya (on the West Anatolian fault line) and also half of the Aegean islands. Additionally, it was observed from Table 9 that four earthquakes ranging between magnitudes 5.5 and 5.9 were associated with the third cluster. To substantiate our findings, we collected data on the main seismic events occurring in the region between the years 2021 and 2024. As indicated in Table 9, numerous earthquakes have occurred within the range of magnitude 4.0 to 4.4. When compared with the LSTM graphs provided in Fig. 16, it is evident that the results accurately reflect reality.

**Table 9:** Frequency distribution of earthquakes by magnitude intervals in the region between 2021 and 2024.

Magnitude (Mwg)	Frequency
3.5–4.0	107
4.1–4.5	24
4.6–5.1	19
5.2–5.6	4

A lot of aftershocks occur together with destructive earthquakes. Therefore, the occurrence of an earthquake with a monthly maximum magnitude of 5 is an indicator of destructive earthquakes. As illustrated in Fig. 16, the upper-bound maximum magnitude estimates obtained from the third cluster reveal the risk of destructive earthquakes from the beginning of 2025 to 2029.

We also compare our findings with some similar studies about the same region. Çam and Duman^101^ conducted a seismic magnitude prediction study for the same region using RNN models other than LSTM. Overall, when evaluating the prediction results, the network accurately predicted earthquakes that would not occur in all regions to a high degree. While the predicted earthquakes that did occur were somewhat accurate, there were also instances of earthquakes that were not predicted or incorrectly predicted. Although the artificial neural network (ANN) generally achieved the desired predictions within certain intervals in the available database, it could not provide highly accurate predictions for earthquakes with a high degree of certainty. Future studies have suggested that incorporating different earthquake parameters into the input parameters used in the models, such as Radon gas density, could lead to more consistent predictions. A similar earthquake magnitude prediction study using LSTM for the region is also attributed to Karcı and Şahin^102^. This study aimed to predict earthquakes based on historical data of earthquakes occurring in Türkiye between 1970 and 2021. Although the dataset used differs, the LSTM model yielded the best predictions, and it was observed that the model selection criteria in our study, obtained through FCM clustering of earthquakes, were more favorable than those of Karcı and Şahin^102^. We also consider that in other studies conducted on same region, the direct utilization of raw catalog data from databases and the filtering out of aftershocks and foreshocks may have influenced the outcome.

Several studies have previously applied clustering methods to earthquake classification and prediction. Novianti et al.^103^, Kamat and Kamath^104^, and Rifa et al.^105^ utilized the k-means clustering algorithm for earthquake classification, while Mirrashid^26^ and Sharma et al.^27^ employed FCM to predict earthquake magnitudes. These approaches have provided valuable insights into seismic activity; however, the proposed methodology in this study enhances the analysis by integrating additional parameters, thus enabling a more comprehensive assessment of both current and potential future seismic activities.

## Conclusion

This study presents a comprehensive and data-driven seismic analysis of earthquakes that occurred in the Western Anatolian Region between 1970 and 2021. By applying the Fuzzy C-Means (FCM) clustering algorithm, earthquakes were classified into three spatially and seismically distinct sub-regions. This approach allowed for the identification of homogeneous seismic zones within a geologically complex setting and provided an alternative to conventional tectonic zoning methods that often rely solely on expert judgment or deterministic fault mapping. Unlike previous studies that focused primarily on historical seismicity statistics or tectonic segmentation (e.g.^2,35^), this study utilized a hybrid methodological framework. By combining unsupervised clustering (FCM), probabilistic modeling (Gumbel distribution), and machine learning (LSTM), it bridges the gap between empirical statistical modeling and physical tectonic interpretation. The monthly maximum earthquake magnitudes of each cluster are then taken into account, and their estimates are obtained with the LSTM algorithm. Since the predicted results obtained here show the monthly maximum earthquake magnitudes, estimates with magnitudes of 3 or 5 show the monthly maximum results. It is predicted that large earthquakes will occur during times when the monthly maximum magnitude is close to 5, as large earthquakes are often accompanied by aftershocks.

This multidisciplinary integration is crucial for developing seismic hazard assessments that are both data-informed and physically meaningful. Each cluster was examined in terms of its spatial extent and seismotectonic characteristics. Cluster 1, covering Istanbul, Izmir, Manisa, and Balıkesir, is located within the Western Anatolian Extensional Province, characterized by active normal faulting and distributed seismicity. The relatively high b-value observed in this cluster reflects the predominance of moderate-magnitude earthquakes and is consistent with the tectonic regime of frequent crustal extension and energy release across a network of grabens (e.g., Gediz, Büyük Menderes). Cluster 2 encompasses regions such as Ankara, Antalya, and Düzce, partially influenced by the Hellenic subduction system, representing a compressional regime with long recurrence intervals and a higher likelihood of large-magnitude earthquakes. The observed low *b*-value in this cluster, coupled with broad magnitude variability, supports this interpretation. However, the hazard function from the Gumbel model showed less frequent but higher-magnitude behavior, aligning with subduction-related seismic patterns reported in prior global studies (e.g.^85^). Cluster 3, including Aydın, Muğla, Crete, and Samos, is transitional between extensional and compressional tectonic zones, making it particularly complex. While the Gutenberg–Richter b-value is intermediate, the Gumbel-based hazard analysis indicated a relatively higher occurrence of moderate-to-large magnitude events within a narrower range, possibly due to the interaction between shallow crustal and deeper subduction-related sources.

One of the novel contributions of this study is the parallel use of two statistical approaches: The *b*-value analysis, which provides long-established insight into seismicity rate and magnitude-frequency distribution and the extreme value modeling via the Gumbel distribution, which focuses on the behavior of extremes and hazard estimation. By interpreting these methods together, the study reveals not just where earthquakes occur, but how magnitudes behave within different tectonic regimes, offering richer insight into regional hazard characteristics.

In the second phase of the study, an LSTM neural network was implemented to predict monthly maximum earthquake magnitudes up to March 2029. The LSTM architecture, which excels at learning long-range temporal dependencies, allowed for mid-term forecasting of seismic behavior. The outputs revealed distinct temporal patterns. Cluster 1 forecasts ranged between 2.0 and 5.7 Mwg, reflecting moderate activity yet retaining potential for large events, particularly in the Istanbul region, which remains a critical area for risk mitigation. Cluster 2 exhibited forecasts between 2.0 and 5.0 Mwg, moderate-magnitude crustal activity. Cluster 3 forecasts ranged from 2.4 to 6.5 Mwg, supporting the idea of persistent, suggesting high and persistent seismic activity. The LSTM analysis findings indicate that the maximum earthquake magnitudes in the 1st region (comprising Istanbul, Izmir, Manisa, Çanakkale, Balıkesir, Edirne) depicted in yellow in Fig. 8b are anticipated to range from 2.0 to 5.7. Conversely, the maximum earthquake magnitudes in the 2nd region (including Konya, Ankara, Antalya, Eskişehir, Bolu, Düzce, İzmit), shown in red in Fig. 8b, are predicted to range from 2.0 to 5.0. Conversely, the maximum earthquake magnitudes in the 3rd region (comprising Aydin, Muğla, Denizli, Burdur, Isparta, Samos, Santorini and Crete) delineated in green in Fig. 8b are projected to range from 2.4 to 6.5. In summary, the most severe earthquakes are expected to occur around Santorini until March 2029.

Unlike earlier LSTM applications that aimed to predict individual earthquake occurrences or binary event classifications (e.g.^106,107^), this study focused on monthly maximums, providing a more reliable indicator of temporal risk build-up rather than isolated predictions. These results emphasize the importance of interpreting seismic hazard not only through conventional geological knowledge or isolated statistical modeling, but through a comprehensive, data-driven framework that integrates physical, statistical, and computational dimensions. In conclusion, the findings indicate that Cluster 3, particularly the half of the Aegean islands region, is likely to experience relatively higher seismic activity in the coming years, both in terms of frequency and magnitude variability. The methodology proposed here is not only applicable to the Aegean region but also scalable and transferable to other seismically active regions of the world. It opens the door to more dynamic and predictive approaches in seismic hazard zoning, earthquake forecasting, and early warning system design. Ultimately, this study demonstrates that meaningful insights into earthquake behavior require both statistical sophistication and geophysical interpretation—a combination that can only be achieved through cross-disciplinary integration. The framework established here serves as a foundational model for next-generation seismic risk analysis in both academic and applied contexts.

Compared to previous studies that rely primarily on historical earthquake catalog statistics or tectonic interpretations alone, this study offers an integrated framework combining clustering analysis, extreme value modeling, and deep learning-based forecasting. This multifaceted approach enables both a better understanding of past seismic behavior and more informed projections of future activity. In conclusion, the results indicate that Cluster 3, particularly the half of the Aegean islands region, is likely to experience relatively higher seismic activity in the coming years. The methodology applied in this study provides a scalable and adaptable model for regional seismic hazard assessment and may serve as a foundation for more dynamic earthquake risk management and early warning strategies.

## Data Availability

The data used in this research were obtained under the Scientific and Technical Research Council of Türkiye (TÜBİTAK) project number 124F059. The datasets used or analyzed in the current study can be obtained from the corresponding author upon reasonable request.
